# Identification of candidate biomarkers correlated with the pathogenesis and prognosis of breast cancer via integrated bioinformatics analysis

**DOI:** 10.1097/MD.0000000000023153

**Published:** 2020-12-04

**Authors:** Shuyu Liu, Xinkui Liu, Jiarui Wu, Wei Zhou, Mengwei Ni, Ziqi Meng, Shanshan Jia, Jingyuan Zhang, Siyu Guo, Shan Lu, Yingfei Li

**Affiliations:** a Department of Clinical Chinese Pharmacy, School of Chinese Materia Medica, Beijing University of Chinese Medicine, Chaoyang District; b Center for Drug Metabolism and Pharmacokinetics Research Research of Herbal Medicines, Institute of Chinese Materia Medica, China Academy of Chinese Medical Sciences, Dongzhimen, Dongcheng District, Beijing, China.

**Keywords:** bioinformatics, biomarker, breast cancer, differentially expressed genes, Gene Expression Omnibus, survival

## Abstract

**Background:**

This study was carried out to identify potential key genes associated with the pathogenesis and prognosis of breast cancer (BC).

**Methods:**

Seven GEO datasets (GSE24124, GSE32641, GSE36295, GSE42568, GSE53752, GSE70947, GSE109169) were downloaded from the Gene Expression Omnibus (GEO) database. Differentially expressed genes (DEGs) between BC and normal breast tissue samples were screened by an integrated analysis of multiple gene expression profile datasets. Hub genes related to the pathogenesis and prognosis of BC were verified by employing protein–protein interaction (PPI) network.

**Results:**

Ten hub genes with high degree were identified, including *CDK1*, *CDC20*, *CCNA2*, *CCNB1*, *CCNB2*, *BUB1*, *BUB1B*, *CDCA8*, *KIF11*, and *TOP2A*. Lastly, the Kaplan–Meier plotter (KM plotter) online database demonstrated that higher expression levels of these genes were related to lower overall survival. Experimental validation showed that all 10 hub genes had the same expression trend as predicted.

**Conclusion:**

The findings of this research would provide some directive significance for further investigating the diagnostic and prognostic biomarkers to facilitate the molecular targeting therapy of BC, which could be used as a new biomarker for diagnosis and to guide the combination medicine of BC.

## 1 Introduction

Breast cancer (BC) is the most frequent cancer among women and is the second leading cause of cancer death among women.^[1]^ According to the American Cancer Society, the 3 most common cancers for women in 2019 are breast, lung, and colorectum, BC alone accounts for 30% of all new cancer diagnoses in women.^[2]^ There is increasing evidence that multiple genes and cellular pathways are involved in the development and progression of BC. Therefore, identifying the progression and significant signaling pathways of disease is critical to discover more effective diagnostic and therapeutic strategies.^[3]^

In recent years, bioinformatics analysis was employed to advance oncology research and lay the foundation for improving disease prevention, early detection, and treatment. The rapid development of this subject enables us to comprehensively screen out the key genes.^[4]^

**Figure 1:**
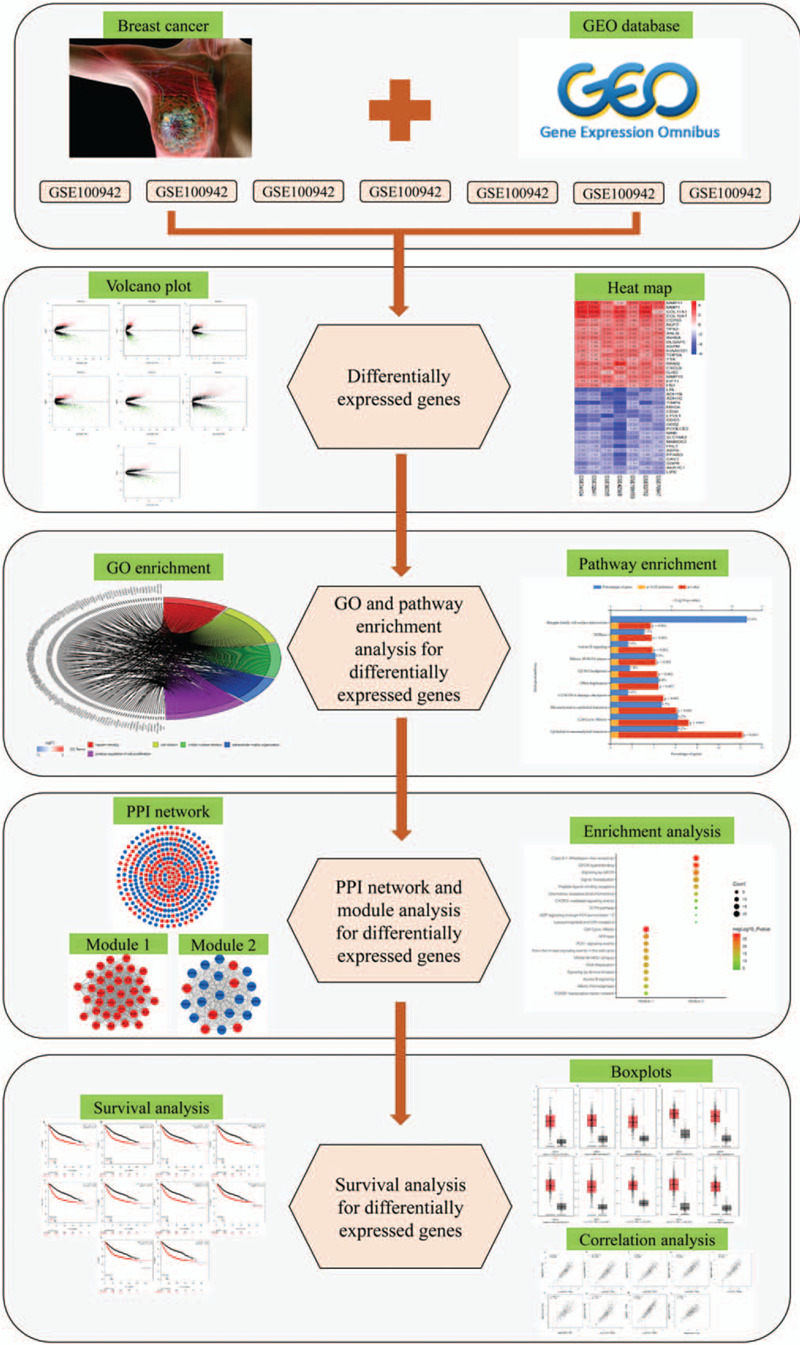
Workflow for identification of hub genes and pathways for BC. BC = breast cancer.

In this study, we tried to detect new indicators of poor prognosis in BC patients and endeavor to provide potential therapeutic targets for this challenging disease. To acquire the differentially expressed genes (DEGs) between BC and healthy breast tissue, bioinformatics methods were used to analyze the gene expression profiling data downloaded from the GEO database. We analyzed DEGs using the limma package with standard data processing. Subsequently, the GO term enrichment analysis and pathway analysis of DEGs were carried out with DAVID database and FunRich software. The protein–protein interaction (PPI) network was then established by STRING and Cytoscape software. Hub genes with high degree of connectivity were picked out subsequently. The Kaplan–Meier (KM) plotter was performed on survival analysis. Accordingly, an integrated analysis of BC on DEGs, will provide further insight into the mechanism of BC. The workflow of this study was shown in Fig. 1.

## 2 Methods

### 2.1 Gene expression profile data

Seven gene expression datasets analyzed in this study (GSE24124, GSE32641, GSE36295, GSE42568, GSE53752, GSE70947, and GSE109169) were screened out based on gene expression omnibus (GEO, http://www.ncbi.nlm.nih.gov/geo/) datasets, a public repository for data storage containing microarray and next-generation sequencing data.^[5]^ Thereafter, datasets were selected for succeeding analysis according to the following criteria: they employed tissue samples gathered from human gastric cancer and normal gastric tissues. They contained at least 30 samples. All the studies on these datasets were published in English language. Limma package in R language was used to analyze the DEGs in BC samples compared with normal samples.^[6]^ Gene integration for the DEGs identified from the 7 datasets was conducted employing RobustRankAggreg.^[7]^ DEGs were obtained according to the criteria: |log_2_FC| ≥1 and adjust *P* value <.05.

### 2.2 Functional enrichment analysis of DEGs

To elucidate potential biological processes and molecular functions with the DEGs, gene ontology (GO) and pathway enrichment analysis were performed utilizing the database for annotation, visualization, and integrated discovery (DAVID, https://david.ncifcrf.gov/) online tool and FunRich software, a software tool used mainly for functional enrichment and interaction network analysis of genes and proteins.^[8,9]^ Meanwhile, *P* value <.05 were defined as the cut-off criteria.

### 2.3 PPI and module analysis

In order to in-deep investigate the PPI information, the protein products of DEGs were matched to the search tool for the retrieval of interacting genes database (STRING, https://string-db.org/cgi/input.pl) to construct a PPI network, and the confidence score ≥0.9 was set as the cutoff criterion. Cytoscape software was used to visualize the resulting PPI network. Subsequently, we selected the hub genes according to connection degree. Moreover, the plug-in molecular complex detection (MCODE) app was applied to detect hub clustering modules in the PPI network.^[10]^ Enrichment analyses for significant modules were also made in subsequent steps.

### 2.4 Survival analysis

The Kaplan–Meier plotter (KM plotter, http://kmplot.com/analysis/) is an online tool used to assess the effect of 54,675 genes on survival using 10,461 cancer samples (1816 ovarian, 5143 breast, 1065 gastric, and 2437 lung cancer). The KM plotter mRNA BC database was applied to estimate the prognosis values of hub genes we had previously identified. According to this software, the relapse free survival (RFS) and overall survival (OS) information were based on GEO, TCGA, and EGA database. The hazard ratio (HR) with 95% confidence intervals and log rank *P* value were calculated to evaluate the association of gene expression with survival and showed on the plot.^[11]^

### 2.5 Analysis of expression level and correlation analysis

The expression level analysis and correlation analysis of the hub genes was carried by the gene expression profiling interactive analysis (GEPIA, http://gepia.cancer-pku.cn/index.html). It provides tumor and normal differential expression analysis so that we could demonstrate the expression of hub genes in BC tissues and normal ones. Then the boxplot was performed to visualize the relationship of these hub genes.^[12]^ Additionally, correlation analysis performs pairwise gene correlation analysis for any given sets of TCGA and/or GTEx expression data and check the relative ratios between 2 genes.^[13]^

### 2.6 Ethical statement

The specimens enrolled in our study were obtained from publicly accessible databases with appropriate consent from institutional review boards at each tissue source site.

## 3 Results

### 3.1 Identification of DEGs

**Figure 2:**
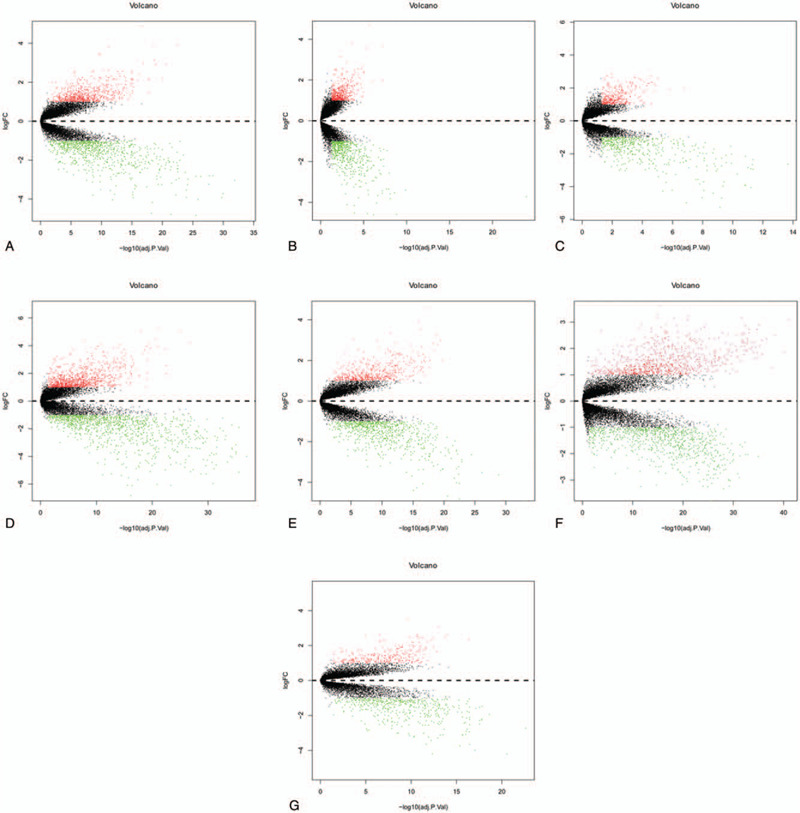
Volcano plot of gene expression profile data in BC samples and normal ones and heat map of DEGs. (A) Volcano plot of GSE24124, (B) volcano plot of GSE32641, (C) volcano plot of GSE36295, (D) volcano plot of GSE42568, (E) volcano plot of GSE53752, (F) volcano plot of GSE70947, (G) volcano plot of GSE109169. BC = breast cancer; DEGs = differentially expressed genes.

**Figure 3:**
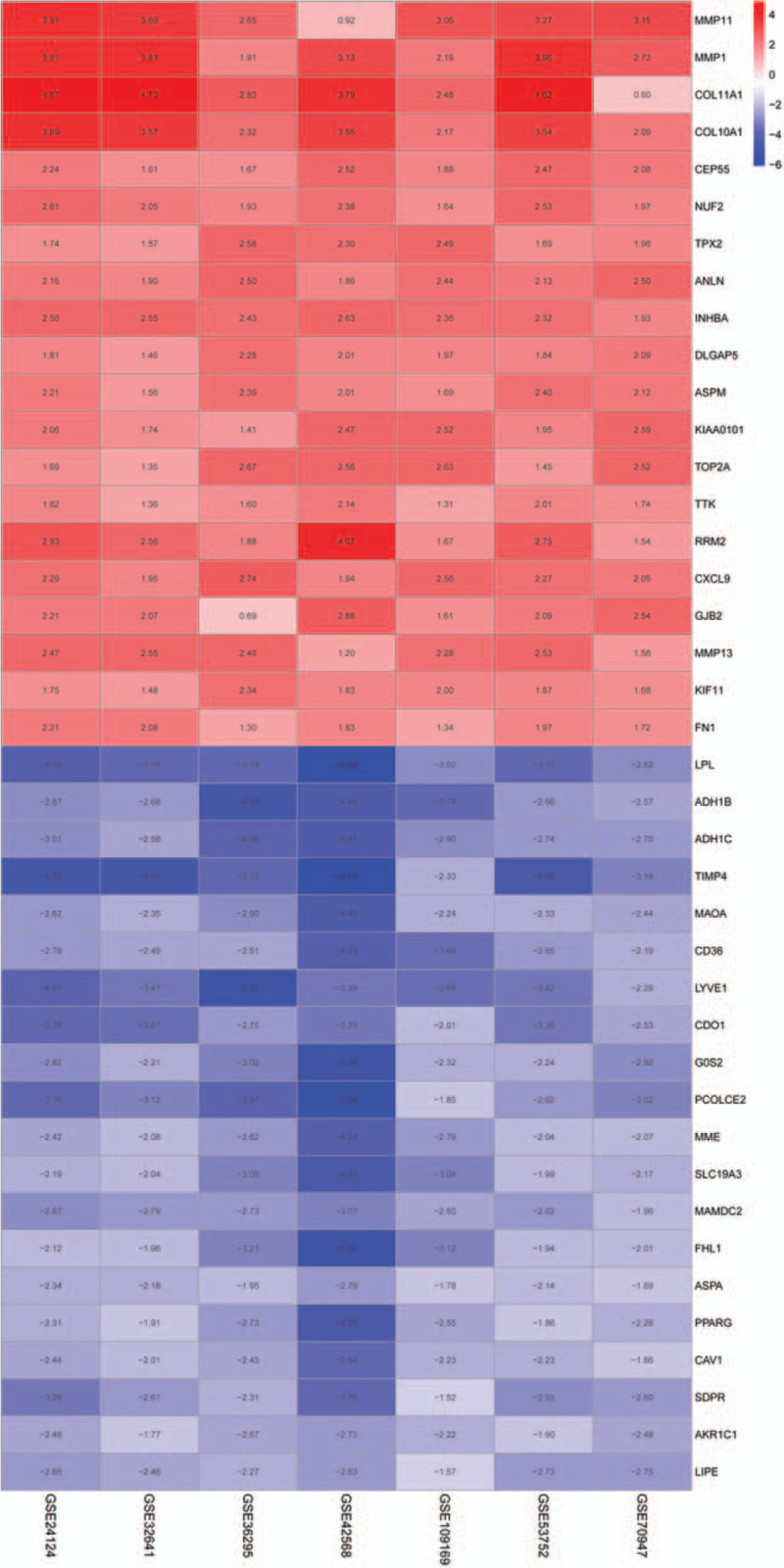
Heat map of DEGs. Blue stands for lower expression levels, red stands for higher expression levels and white represents no different expression among these genes. Each column expresses one dataset and each row is one gene. The number in each rectangle means the normalized gene expression level. The gradual color ranged from green to red shows the changing process from down-regulation to up-regulation. DEGs = differentially expressed genes.

The detailed information of the samples in the included datasets was shown in Table 1. A total of 676 DEGs comprising 445 down-regulated and 231 up-regulated genes were retrieved after the integrated analysis of seven GEO datasets (see Table S1, Supplemental Content, which shows the information for 231 up-regulated genes and 445 down-regulated genes). The DEGs are shown in the volcano plot (Fig. 2). Figure 3 showed top 20 down-regulated and up-regulated genes in the integrated microarray analysis.

**Table 1:** Information for the 7 GEO datasets included in the current study.

Dataset	Reference	Platform	Number of samples (tumor/control)
GSE24124	Liu et al, 2014	GPL887-Agilent-012097 Human 1A Microarray (V2) G4110B (Feature Number version)	119 (99/20)
GSE32641	Liu et al, 2014	GPL887-Agilent-012097 Human 1A Microarray (V2) G4110B (Feature Number version)	102 (95/7)
GSE36295	Manikandan J et al, 2018	GPL6244-[HuGene-1_0-st] Affymetrix Human Gene 1.0 ST Array [transcript (gene) version]	50 (45/5)
GSE42568	Clarke C et al, 2019	GPL570-[HG-U133_Plus_2] Affymetrix Human Genome U133 Plus 2.0 Array	121 (104/17)
GSE53752	Kuo et al, 2014	GPL7264-Agilent-012097 Human 1A Microarray (V2) G4110B (Probe Name version)	76 (51/25)
GSE70947	Quigley et al, 2018	GPL13607-Agilent-028004 SurePrint G3 Human GE 8x60K Microarray (Feature Number version)	296 (148/148)
GSE109169	Chang et al, 2019	GPL5175-[HuEx-1_0-st] Affymetrix Human Exon 1.0 ST Array [transcript (gene) version]	50 (25/25)

GO = Genome Ontology.

### 3.2 Functional enrichment analysis of DEGs

**Figure 4:**
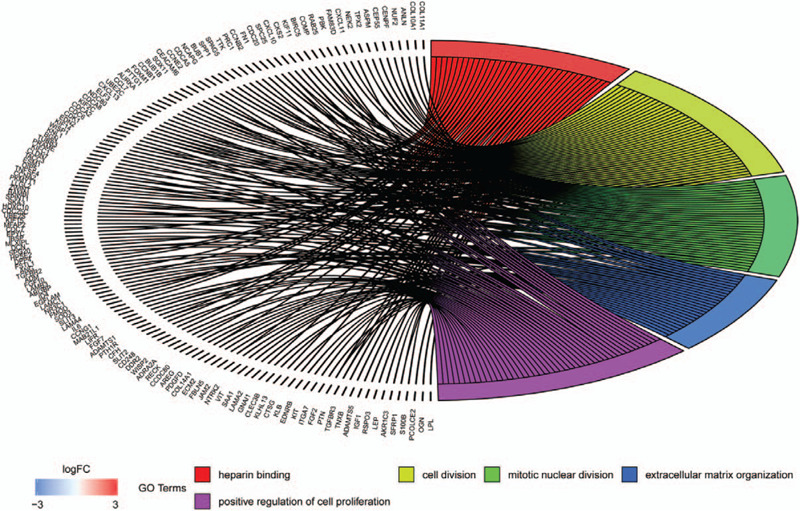
GO enrichment analysis for DEGs. DEGs = differentially expressed genes; GO = Genome Ontology.

**Figure 5:**
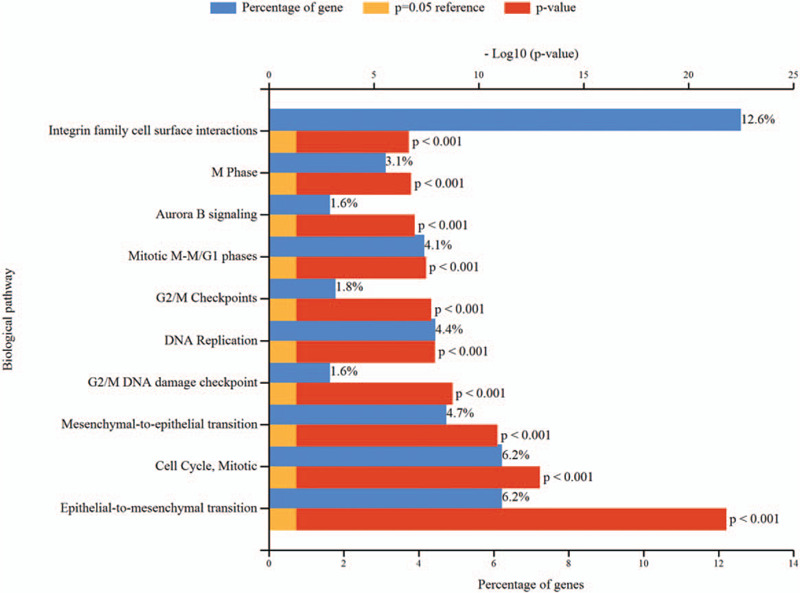
Biological pathway for DEGs. DEGs = differentially expressed genes.

Enrichment analyses for the up-regulated and down-regulated DEGs after gene integration were performed via DAVID and FunRich software. GO analysis results showed that up-regulated DEGs and down-regulated DEGs were particularly enriched in biological processes (BPs), including cell division, mitotic nuclear division, extracellular matrix organization, and positive regulation of cell proliferation. Moreover, the GO MFs analysis mainly involved in heparin binding (Fig. 4 and Table S2, Supplemental Content, which shows the information of GO enrichment analysis). The results of pathway enrichment analysis showed that DEGs were mainly enriched in pathways in epithelial-to-mesenchymal transition, cell Cycle [Mitotic], mesenchymal-to-epithelial transition, G2/M DNA damage checkpoint, DNA Replication, G2/M Checkpoints, mitotic M-M/G1 phases, aurora B signaling, M phase, and integrin family cell surface interactions (Fig. 5 and Table S3, Supplemental Content, which shows the information for pathway enrichment analysis).

### 3.3 PPI and module analysis

**Figure 6:**
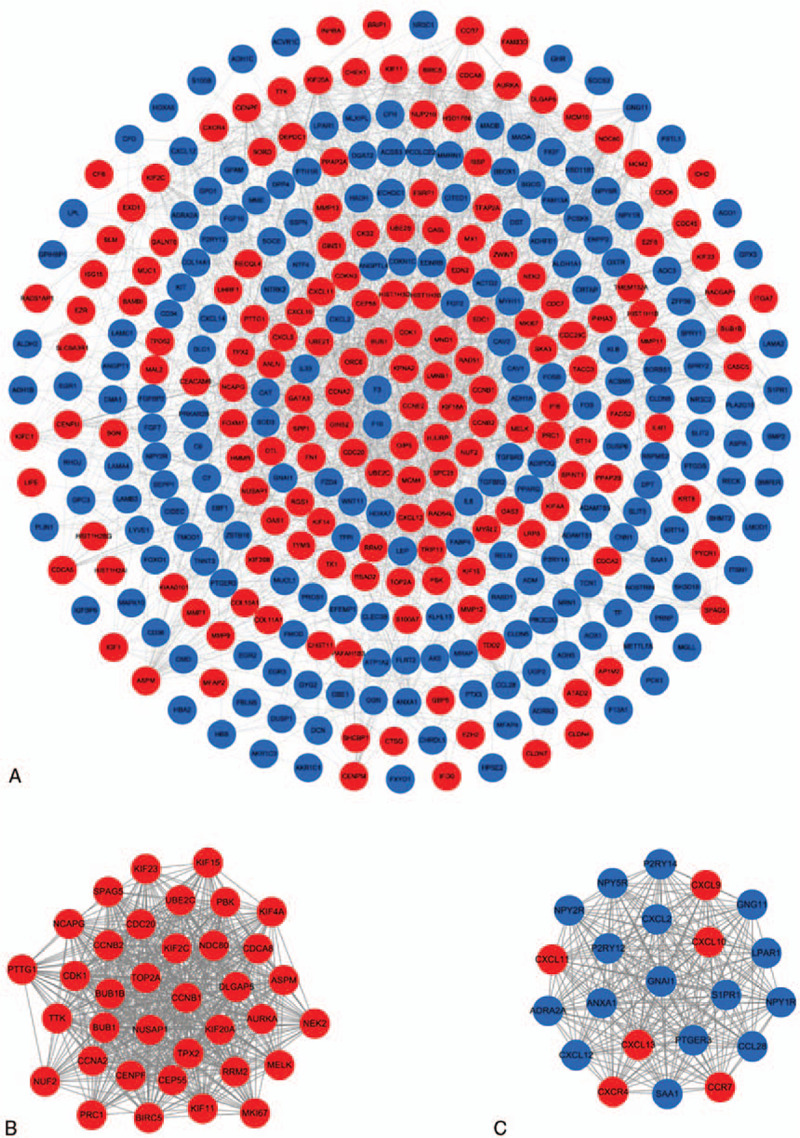
PPI network and hub clustering modules. (A) The PPI network of overlapping DEGs. (B) Module 1 (MCODE score = 32.971). (C) Module 2 (MCODE score = 22.000). Blue circles represent down-regulated genes and red circles represent up-regulated genes. DEGs = differentially expressed genes; PPI = protein–protein interaction.

**Figure 7:**
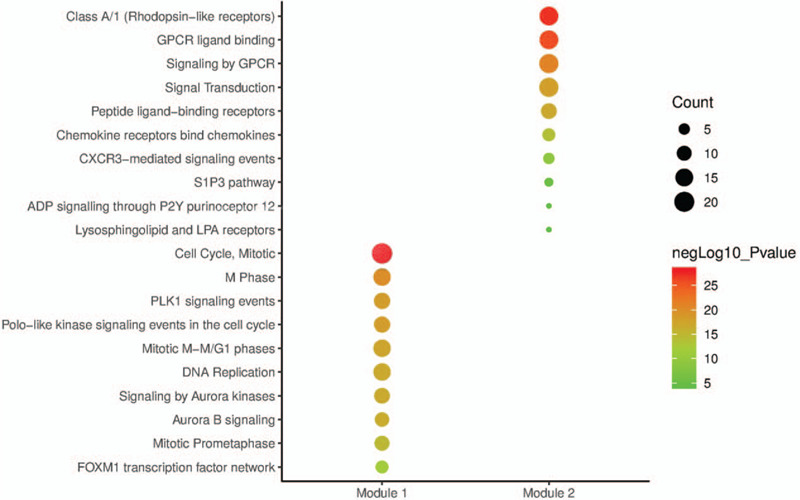
Pathway enrichment analysis of the DEGs in the 2 modules. The *y*-axis shows significantly enriched pathways, and the *x*-axis shows different gene categories. DEGs = differentially expressed genes.

A total of 364 nodes and 1819 edges were mapped in the PPI network (Fig. 6A and Table S4, Supplemental Content, which shows the information for PPI network). Combining the degree scores of the PPI network yielded a total of 10 genes with higher degree scores, which were then selected as hub genes, namely, Cyclin-dependent kinase 1 (*CDK1*), Cell division cycle protein 20 homolog (*CDC20*), Cyclin-A2 (*CCNA2*), G2/mitotic-specific cyclin-B1 (*CCNB1*), G2/mitotic-specific cyclin-B2 (*CCNB2*), Mitotic checkpoint serine/threonine-protein kinase BUB1 (*BUB1*), Mitotic checkpoint serine/threonine-protein kinase BUB1 beta (*BUB1B*), Borealin (*CDCA8*), DNA topoisomerase II alpha (*TOP2A*), and Kinesin-like protein KIF11 (*KIF11*) (Table 2). All of these 10 genes were of high expression in BC. Additionally, in order to detect significant clustering modules in this PPI network we performed module analysis and obtained top 2 modules with high scores. As shown in the picture, all of the 10 hub genes were contained in module 1 (Fig. 6B and C). At the aspect of pathway enrichment analysis, module 1 was closely correlated with Cell Cycle (Mitotic), M Phase, PLK1 signaling events, Polo-like kinase signaling events in the cell cycle, Mitotic M-M/G1 phases, DNA Replication, Signaling by Aurora kinases, Aurora B signaling, Mitotic Prometaphase, and FOXM1 transcription factor network; module 2 was highly connected to Class A/1 (Rhodopsin-like receptors), GPCR ligand binding, Signaling by GPCR, Signal Transduction, Peptide ligand-binding receptors, Chemokine receptors bind chemokines, CXCR3-mediated signaling events, S1P3 pathway, ADP signaling through P2Y purinoceptor 12 and Lysosphingolipid, and LPA receptors (Fig. 7 and Table S5, Supplemental Content, which shows the information for module 1 and module 2).

**Table 2:** Hub genes with high degree of connectivity.

Gene	Degree	Type	MCODE cluster
CDK1	75	up	Module 1
CDC20	63	up	Module 1
CCNA2	60	up	Module 1
CCNB1	60	up	Module 1
CCNB2	59	up	Module 1
BUB1	58	up	Module 1
BUB1B	53	up	Module 1
CDCA8	52	up	Module 1
TOP2A	51	up	Module 1
KIF11	50	up	Module 1

BUB1 = mitotic checkpoint serine/threonine-protein kinase BUB1; BUB1B = mitotic checkpoint serine/threonine-protein kinase BUB1 beta; CCNA2 = Cyclin-A2; CCNB1 = G2/mitotic-specific cyclin-B1; CCNB2 = G2/mitotic-specific cyclin-B2; CDC20 = cell division cycle protein 20 homolog; CDCA8 = Borealin; CDK1 = cyclin-dependent kinase 1; KIF11 = Kinesin-like protein KIF11; MCODE = molecular complex detection; TOP2A = DNA topoisomerase II alpha.

### 3.4 Survival analysis

**Figure 8:**
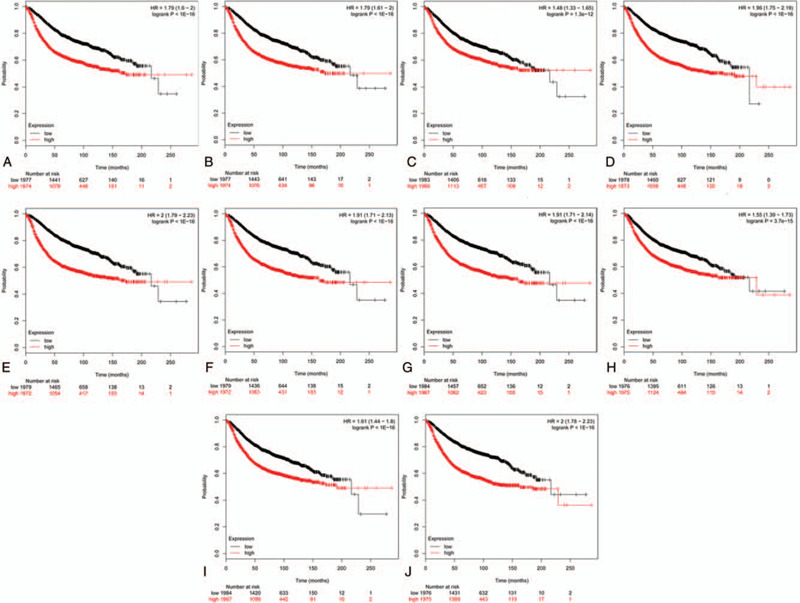
Prognostic roles of ten hub genes in the BC patients. Survival curves are plotted for BC cancer patients. (A) BUB1; (B) BUB1B; (C) CCNA2; (D) CCNB1; (E) CCNB2; (F) CDC20; (G) CDCA8; (H) CDK1; (I) KIF11; (J) TOP2A. BC = breast cancer; BUB1 = mitotic checkpoint serine/threonine-protein kinase BUB1; BUB1B = mitotic checkpoint serine/threonine-protein kinase BUB1 beta; CCNA2 = Cyclin-A2; CCNB1 = G2/mitotic-specific cyclin-B1; CCNB2 = G2/mitotic-specific cyclin-B2; CDC20 = cell division cycle protein 20 homolog; CDCA8 = Borealin; CDK1 = cyclin-dependent kinase 1; KIF11 = Kinesin-like protein KIF11; TOP2A = DNA topoisomerase II alpha.

**Figure 9:**
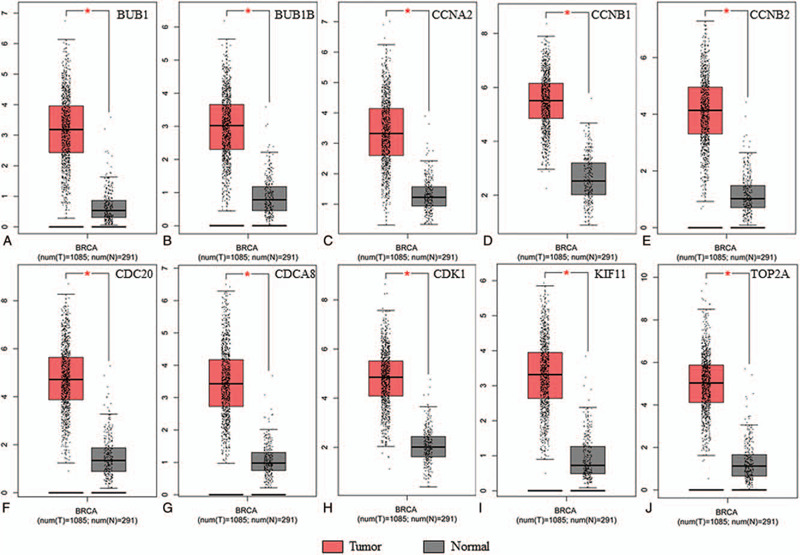
Analysis of ten hub genes expression level in human BC. The red and gray boxes represent cancer and normal tissues, respectively. (A) BUB1; (B) BUB1B; (C) CCNA2; (D) CCNB1; (E) CCNB2; (F) CDC20; (G) CDCA8; (H) CDK1; (I) KIF11; (J) TOP2A. BC = breast cancer; BUB1 = mitotic checkpoint serine/threonine-protein kinase BUB1; BUB1B = mitotic checkpoint serine/threonine-protein kinase BUB1 beta; CCNA2 = Cyclin-A2; CCNB1 = G2/mitotic-specific cyclin-B1; CCNB2 = G2/mitotic-specific cyclin-B2; CDC20 = cell division cycle protein 20 homolog; CDCA8 = Borealin; CDK1 = cyclin-dependent kinase 1; KIF11 = Kinesin-like protein KIF11; TOP2A = DNA topoisomerase II alpha.

**Figure 10:**
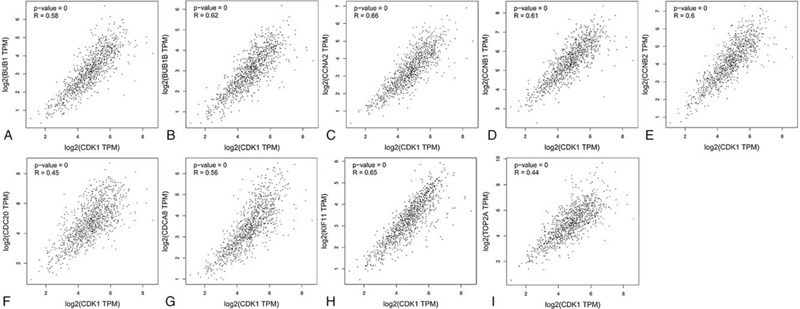
Correlation analysis of 9 hub genes and CDK1 in BC. BC = breast cancer; CDK1 = cyclin-dependent kinase 1.

To investigate the prognostic values of the 10 potential hub genes, the survival analysis was conducted by the K–M plotter platform. Coincidentally, Fig. 8 showed the K–M survival curves for the 10 hub genes, including *CDK1* (HR = 1.55, *P* = 3.7E–15), *CCNB1* (HR = 1.96, *P* < 1E–16), *CCNB2* (HR = 2, *P* < 1E–16), *CCNA2* (HR = 1.48, *P* = 1.3E–12), *BUB1* (HR 1.79, *P* < 1E–16), *BUB1B* (HR = 1.79, *P* < 1E–16), *CDCA8* (HR = 1.91, *P* < 1E–16), *CDC20* (HR 1.91, *P* < 1E–16), *KIF11* (HR = 1.61, *P* < 1E–16) and *TOP2A* (HR = 2, *P* < 1E–16). It was found that all the 10 genes were risky genes for prognosis with HR > 1 and *P* < .01, which suggested the significant value and reliability of our bioinformatics analysis in discovering hub genes in BC. Obviously, higher expression of the 10 genes predicted shorter survival times for BC patients. Then, the GEPIA tool was applied to catch the expression level of the hub genes between BC tissues and normal ones. Interestingly, the result reflected that all the 10 genes were highly expressed in BC tissues compared with normal tissues (Fig. 9). Therefore, these genes might not only play a vital role in influencing the prognosis of BC patients but also be crucial in the pathology and progression of this disease. Because *CDK1* possessed the highest degree value in the PPI network constructed in the above step, this gene was chosen for conducting correlation analysis. The result showed that *CDK1* had strongly high correlation with all the other 9 hub genes (Pearson *r* > 0.4 and *P* < .01), in which the top 3 genes that were positively correlated with *CDK1* were *CCNA2*, *KIF11*, and *BUB1B* and the Pearson correlation coefficients were 0.66, 0.65, and 0.62, respectively (Fig. 10). These results highlighted the importance of *CDK1* as the hub gene in the PPI network and in the interaction with other disease-related genes in BC.

## 4 Discussion

BC is the most common invasive cancer in women globally and affects about 12% of women worldwide.^[14]^ Despite advances in screening and detection, BC remains a momentous threat to women because of the rapid development of drug resistance and a lack of novel therapeutic targets and agents. Hence, specific and sensitive biomarkers of BC are urgently to be detected.

This study investigated 7 microarray datasets from the GEO database. A total of 676 DEGs between BC and normal samples were screened, comprising 231 up-regulated and 445 down-regulated genes. Since BC has a high mortality rate, early molecular diagnosis plays an important role achieving a favorable prognosis. Until now, numerous genes have been found to participate in BC formation and can serve as specific diagnostic indicators with potential clinical applications. Therefore, it is of great importance to identify more candidate genes for the diagnosis and treatment of BC.

GO function and pathway enrichment analysis were performed to further analyze the mechanisms of action of these DEGs. These DEGs were associated with the GO BP terms such as cell division, mitotic nuclear division, extracellular matrix organization, positive regulation of cell proliferation, response to drug, extracellular matrix disassembly, and response to glucocorticoid, and significantly enriched in heparin binding and oxidoreductase activity as MF terms. Furthermore, the pathways of DEGs were mainly enriched in epithelial-to-mesenchymal transition, cell Cycle (Mitotic), mesenchymal-to-epithelial transition, G2/M DNA damage checkpoint, DNA Replication, G2/M Checkpoints, mitotic M-M/G1 phases, aurora B signaling, M phase and integrin family cell surface interactions. Among these DEGs, 10 hub genes with high degree of connectivity were selected in the PPI network, namely, *CDK1*, *CDC20*, *CCNA2*, *CCNB1*, *CCNB2*, *BUB1*, *BUB1B*, *CDCA8*, *KIF11*, and *TOP2A*. The survival analysis found that higher expression of all the 10 genes predicted more worse prognosis for BC patients and coincidentally all of them were overexpressed in BC tissues compared with normal tissues, which suggested that these genes might not only play a vital role in the prognosis of BC patients but also be crucial in the pathology and progression of this disease. Thus, these 10 genes were identified as hub genes correlated with the pathogenesis and prognosis of BC in our study, and they were detailedly discussed as follows.

Cyclin-dependent kinase 1 also known as CDK1, it is a key player in cell cycle regulation.^[15]^ CDK1 is the central mammalian regulator of cell proliferation and a promising therapeutic target for BC. In breast tumors from patients, currently research found a negative correlation between CDK1 accumulation and βTrCP levels, and a positive correlation with the degree of tumor malignancy.^[16]^ BC cells displayed a wide range of sensitivity to siRNA-mediated MYC knockdown, and the sensitivity was correlated with MYC protein expression and MYC phosphorylation level. Kang et al^[17]^ has reported that the apoptosis and reduced viability of MYC-dependent cells was significantly induced by the inactivation of CDK1. These results indicated that further investigation of CDK1 inhibition as a potential therapy for MYC-dependent BC is warranted.

Cell-division cycle protein 20, an essential regulator of cell division, is encoded by the CDC20 gene.^[18,19]^ CDC20 serves as a regulatory protein interacting with many other proteins at multiple points in the cell cycle. It also destructs S and M-phase (S/M) cyclins to inactivates S/M cyclin-dependent kinases (Cdks) and allows the cell to exit from mitosis.^[20]^ Consistent with the notion that CDC20 may function as an oncoprotein, recent studies have shown that CDC20 is highly expressed in various types of human tumors. Jiang et al^[21]^ reported that CDC20 is over-expressed in BC cells compared with normal mammary epithelial cells. Karra et al^[22]^ reported the association of high CDC20 immune expression with terribly poor outcome of BC patients, which indicates that CDC20 is promising candidate for clinical applications in BC prognostication, especially in the challenging prognostic decisions of triple negative breast cancer (TNBC). Taken together, CDC20 is often over-expressed in a majority of human cancers, supporting its oncogenic role in promoting tumorigenesis, and thus CDC20 is a legitimate target of drug development for the treatment of human malignancies.^[23]^

Cyclin-A2 is a protein encoded by the CCNA2 gene.^[24]^ It is a member of the cyclin family. In particular, Cyclin-A2 can activate 2 different CDK kinases; it binds CDK2 during S phase, and CDK1 during the transition from G2 to M phase.^[25]^ Apart from BC, increased expression of cyclin-A2 has also been detected in many types of cancer such as cervical, liver, and lung.^[26–30]^ Although it is not clear whether increased expression of cyclin-A2 is a cause or result of tumorigenesis, it is indicative of prognostic values such as predictions of survival or relapse.^[26]^ Overexpression of cyclin-A2 in mammalian cells can lead to the delayed onset of metaphase and anaphase.^[31]^ It is also likely that cyclin-A2-CDK is partly responsible for tumorigenesis by the phosphorylation of oncoproteins or tumor suppressors like p53.^[32]^

Cyclin-B1 is a regulatory protein involved in mitosis. Previous studies reported that high cyclin-B1 expression levels are found in diversity of cancers such as breast, cervical, gastric, colorectal, head and neck squamous cell, non-small-cell lung cancer, and others.^[33–37]^ High expression levels are usually detected before the tumor cells become immortalized and aneuploid which can contribute to the aggressive nature of certain cancers.^[38]^ These high levels of cyclin-B1 can also be associated to the extent of tumor aggressiveness, therefore, concentration of cyclin-B1 can be used to ascertain the prognosis of cancer patients.^[34,39]^ It has been proved that an increase in expression of cyclin-B1/cdc2 is significantly higher in breast tumor tissue, which can be used as a tool to determine prognosis of patients with BC.^[34,40]^ In early stages of cancer, cyclin-B1 is usually recognized by the immune system with its high concentration, leading to the production of antibodies and T cells. Then it would be possible to monitor the immune response for early cancer detection.^[41]^

Cyclin-B2 belongs to the cyclin family and is essential component of the cell cycle regulatory machinery.^[42]^ In accord with a vital role in cell growth, numerous studies detected overexpression of CCNB2 in human tumors, including lung, pituitary adenomas, and colorectal adenocarcinoma.^[43–46]^ Shubbar et al^[4]^ used multivariate Cox regression analysis designated that CCNB2 protein expression is an independent prognostic marker of disease-specific survival (DSS) in BC. This result suggests that cytoplasmic CCNB2 may serve as an oncogene and could function as a potential biomarker of unfavorable prognosis over short-term follow-up in BC.

Mitotic checkpoint serine/threonine-protein kinase BUB1 also known as BUB1, it is an enzyme that is encoded by the BUB1 gene in humans.^[47,48]^ Disturbed mitotic checkpoints are a common feature of numerous human cancers. More exactly, mutations in the spindle checkpoint can result in chromosomal instability and aneuploidy, a feature exhibit in over 90% of all solid tumors.^[49]^ In this study, over expression of BUB1 gene in BC patients has been identified. However, loss-of-function mutations or reduced gene expression of BUB1 have been detected in several human tumors as colon, gastric, esophageal, melanoma, and BC.^[50]^ Research suggests that there is a correlation between BUB1 expression levels and the localization of tumors along with their severity. For example, low BUB1 expression levels resulted in more lymphomas, sarcomas, and lung tumors, whereas higher ones led to sarcomas and tumors in the liver.^[51]^ An animal experiments indicated that BUB1 possibly involved in tumorigenesis, where mice with reduced BUB1 expression appeared an increase in tumor susceptibility.^[52,53]^ Han et al^[54–57]^ found that BUB1 expression is correlated with a poor clinical prognosis in patients with BC. They depleted BUB1 using shRNAs reduces cancer stem cell potential of the MDA-MB-231 BC cell line, leading to inhibited formation of xenografts in immunocompromised mice. These results show that BUB1 may be linked with cancer stem cell potential and could be a target for developing anti-BC stem cell therapies. In addition, mitotic checkpoint serine/threonine-protein kinase BUB1 beta is an enzyme that is encoded by the BUB1B gene.^[58]^ This gene encodes a kinase involved in spindle checkpoint function and chromosome segregation.^[59]^ Incidentally, impaired spindle checkpoint function has been found in a variety of cancer, which may have a potential relationship with BUBIB.^[47]^

Borealin is a protein encoded by the CDCA8 gene.^[60–62]^ CDCA8 can function as a possible oncogene that may be up-regulated in multifarious types of cancer, which will cause the incidence of various cancers, such as bladder cancer, gastric cancer, lung cancer, and cutaneous melanoma.^[63–65]^ As an essential regulator of mitosis, researches indicate that CDCA8 is found to be especially over-expressed in TNBC,^[66]^ and has a high expression level in male BC, invasive lobular BC, and invasive ductal BC subtypes.^[67]^ The KM plotter analysis carried out by Bu et al^[68]^ indicated that higher CDCA8 expression was positively associated with poor prognosis with a probability lower than 0.4 at the 5-year interval (*P* = .035), suggesting that CDCA8 is a critical mediator of estrogen-stimulated BC cell growth and survival, which can be identified as a novel target in BC treatment. Phan et al^[67]^ analyzed the mRNA expression of the CDCA genes as related to BC patient survival. This study reported that CDCA3, CDCA5, and CDCA8 mRNA expression levels were significantly higher than the control sample in both clinical tumor sample and cancer cell lines, which dramatically reduced patient survival. Besides, CDCA8 expression was also reported to be correlated with BUB1 and CCNB1.

Kinesin family member 11 is also known as Kinesin-5, which has been widely studied for its role in mitosis and the potential as a therapeutic target in the treatment of cancer.^[69]^ Inhibitors of KIF11 have been developed as chemotherapeutic agents for cancer treatment.^[70]^ Pei et al^[71]^ indicated that KIF11 contributes to the progression and prognosis of human BC. Their study suggested a significant association between the up-regulation of KIF11 expression and the progression of BC, KIF11 up-regulation represents an independent prognostic indicator for the survival of patients with BC. This conclusion coincide with the analysis of the relationship between KIF11 expression and the survival time of patients with BC in The Cancer Genome Atlas database indicated that overexpressed KIF11 occurred frequently in BC and was associated with a poor prognosis.^[72]^

Topoisomerase II alpha [TOP2A] is one of the 2 types of Type II topoisomerases.^[73,74]^ High expression of TOP2A is detected in several types of cancer, and TOP2A has been recognized as a cancer target in clinical application.^[75–77]^ It is well known that the HER2 gene has a well-established biological and clinical role in BC, and the HER2 amplicon on chromosome 17 harbors a great quality of genes involved in BC pathophysiology. TOP2A is one of the genes closely related to HER2 and its protein product, topoisomerase II α, is the molecular target of anthracycline treatment.^[78]^ Brase et al^[79]^ showed that TOP2A RNA is a powerful prognostic marker in BC and is also connected with a favorable response to anthracyclin-based therapy.

However, there were still several limitations of the present study. First, as a result of our study only focused on the genes that are usually identified as significant changes in multiple data sets, there is no consideration of other characteristics like age, sex, tumor staging, and classification in detail. Thus, larger sample sizes are necessary to verify the findings of this study. Second, more experiments, for example chemistry experiments comparing expression in BC tissues and normal tissues, should be conducted to confirm the target genes.

## 5 Conclusion

In conclusion, our bioinformatics analysis identified that *CDK1*, *CDC20*, *CCNA2*, *CCNB1*, *CCNB2*, *BUB1*, *BUB1B*, *CDCA8*, *KIF11*, and *TOP2A* might be core genes related to BC. However, further experiments are required to confirm these predictions in BC. We hope this study may provide some evidence for the future genomic individualized treatment of BC from new perception.

## Author contributions

**Conceptualization:** Shuyu Liu, Xinkui Liu.

**Data curation:** Shuyu Liu, Xinkui Liu.

**Formal analysis:** Shuyu Liu, Xinkui Liu.

**Funding acquisition:** Jiarui Wu.

**Investigation:** Shuyu Liu, Xinkui Liu.

**Methodology:** Shuyu Liu, Xinkui Liu.

**Project administration:** Xinkui Liu, Jiarui Wu.

**Software:** Xinkui Liu.

**Supervision:** Xinkui Liu, Jiarui Wu.

**Validation:** Mengwei Ni.

**Visualization:** Shuyu Liu, Xinkui Liu, Wei Zhou, Ziqi Meng, Shanshan Jia, Jingyuan Zhang, Siyu Guo, Shan Lu, YingFei Li.

**Writing – original draft:** Shuyu Liu, Xinkui Liu.

**Writing – review & editing:** Xinkui Liu.

## Supplementary Material

Supplemental Digital Content

## Supplementary Material

Supplemental Digital Content

## Supplementary Material

Supplemental Digital Content

## Supplementary Material

Supplemental Digital Content

## Supplementary Material

Supplemental Digital Content
