# Supplementary material for: Identification of candidate biomarkers correlated with the pathogenesis and prognosis of breast cancer via integrated bioinformatics analysis
Source: Medicine (Baltimore). 2020 Dec 4;99(49):e23153. doi: 10.1097/MD.0000000000023153 (PMC7717725; doi:10.1097/MD.0000000000023153)
Supplement: Supplemental Digital Content [file medi-99-e23153-s001.docx]

Table S1. Information for 231 up-regulated genes and 445 down-regulated genes.

| Name | Type | *P* value | adj*P* value | LogFC |
| --- | --- | --- | --- | --- |
| ADAMDEC1 | up-regulated | 1.02E-10 | 2.72E-06 | 1.888707 |
| AEBP1 | up-regulated | 5.24E-07 | 0.014001691 | 1.052418 |
| AIM2 | up-regulated | 3.97E-08 | 0.001061395 | 1.135345 |
| ANKRD22 | up-regulated | 9.08E-07 | 0.024263138 | 1.220292 |
| ANLN | up-regulated | 1.45E-15 | 3.87E-11 | 2.212275 |
| AP1M2 | up-regulated | 2.05E-09 | 5.49E-05 | 1.524077 |
| APOBEC3B | up-regulated | 2.49E-08 | 0.000666743 | 1.374379 |
| ASPM | up-regulated | 5.53E-15 | 1.48E-10 | 2.053419 |
| ATAD2 | up-regulated | 4.15E-12 | 1.11E-07 | 1.502009 |
| ATP2C2 | up-regulated | 7.96E-09 | 0.000212684 | 1.076816 |
| AURKA | up-regulated | 5.57E-11 | 1.49E-06 | 1.485676 |
| BAIAP2L1 | up-regulated | 2.45E-07 | 0.006548497 | 1.047009 |
| BAMBI | up-regulated | 1.47E-07 | 0.003932577 | 1.119346 |
| BGN | up-regulated | 1.90E-11 | 5.07E-07 | 1.286468 |
| BIRC5 | up-regulated | 3.88E-10 | 1.04E-05 | 1.867124 |
| BLM | up-regulated | 1.03E-09 | 2.76E-05 | 1.143224 |
| BRI3BP | up-regulated | 8.97E-08 | 0.002397468 | 1.038734 |
| BRIP1 | up-regulated | 7.41E-07 | 0.019810966 | 1.099563 |
| BUB1 | up-regulated | 5.64E-11 | 1.51E-06 | 1.685651 |
| BUB1B | up-regulated | 2.41E-08 | 0.000643992 | 1.59851 |
| C11orf80 | up-regulated | 2.62E-09 | 7.00E-05 | 1.109805 |
| C15orf48 | up-regulated | 1.49E-07 | 0.003984345 | 1.606661 |
| C1orf106 | up-regulated | 1.24E-06 | 0.033207086 | 1.456072 |
| CALML5 | up-regulated | 2.04E-07 | 0.005458036 | 1.128368 |
| CAPS | up-regulated | 1.07E-06 | 0.028674801 | 1.21192 |
| CCL7 | up-regulated | 2.81E-08 | 0.00075231 | 1.481388 |
| CCNA2 | up-regulated | 8.78E-09 | 0.000234722 | 1.223445 |
| CCNB1 | up-regulated | 6.36E-12 | 1.70E-07 | 1.530402 |
| CCNB2 | up-regulated | 2.42E-11 | 6.48E-07 | 1.730373 |
| CCNE2 | up-regulated | 2.94E-12 | 7.87E-08 | 1.635109 |
| CCR7 | up-regulated | 1.09E-07 | 0.002911942 | 1.186209 |
| CDC20 | up-regulated | 8.07E-13 | 2.16E-08 | 1.780386 |
| CDC25C | up-regulated | 7.70E-08 | 0.002058537 | 1.084216 |
| CDC45 | up-regulated | 7.77E-09 | 0.000207746 | 1.174854 |
| CDC6 | up-regulated | 2.07E-09 | 5.53E-05 | 1.373366 |
| CDC7 | up-regulated | 5.43E-07 | 0.014514621 | 1.016757 |
| CDCA2 | up-regulated | 2.89E-09 | 7.74E-05 | 1.420876 |
| CDCA5 | up-regulated | 9.24E-10 | 2.47E-05 | 1.671815 |
| CDCA8 | up-regulated | 9.32E-11 | 2.49E-06 | 1.435076 |
| CDK1 | up-regulated | 9.73E-08 | 0.002600985 | 1.365521 |
| CDKN3 | up-regulated | 1.53E-10 | 4.10E-06 | 1.409757 |
| CEACAM6 | up-regulated | 5.60E-09 | 0.000149742 | 1.630458 |
| CENPF | up-regulated | 2.01E-13 | 5.37E-09 | 2.125111 |
| CENPM | up-regulated | 2.95E-07 | 0.007888569 | 1.267572 |
| CENPU | up-regulated | 6.36E-08 | 0.001699386 | 1.100782 |
| CEP55 | up-regulated | 1.80E-16 | 4.80E-12 | 2.067226 |
| CFB | up-regulated | 1.56E-08 | 0.000416597 | 1.234499 |
| CHEK1 | up-regulated | 1.30E-09 | 3.49E-05 | 1.348898 |
| CHST11 | up-regulated | 6.10E-08 | 0.001629272 | 1.156452 |
| CKS2 | up-regulated | 1.47E-11 | 3.93E-07 | 1.823002 |
| CLDN4 | up-regulated | 2.91E-07 | 0.007780516 | 1.181681 |
| CLDN7 | up-regulated | 3.69E-09 | 9.87E-05 | 1.257207 |
| COL10A1 | up-regulated | 2.57E-17 | 6.86E-13 | 3.018861 |
| COL11A1 | up-regulated | 3.24E-18 | 8.66E-14 | 3.412398 |
| COMP | up-regulated | 1.05E-11 | 2.81E-07 | 1.920887 |
| CTHRC1 | up-regulated | 2.84E-10 | 7.60E-06 | 1.544969 |
| CXCL10 | up-regulated | 3.87E-10 | 1.03E-05 | 1.82146 |
| CXCL11 | up-regulated | 8.73E-11 | 2.33E-06 | 1.991534 |
| CXCL13 | up-regulated | 1.56E-10 | 4.17E-06 | 1.461873 |
| CXCL9 | up-regulated | 1.25E-14 | 3.34E-10 | 2.25548 |
| CXCR4 | up-regulated | 2.40E-10 | 6.41E-06 | 1.117139 |
| CYB561 | up-regulated | 3.45E-11 | 9.21E-07 | 1.22386 |
| DEPDC1 | up-regulated | 1.99E-08 | 0.000531936 | 1.373643 |
| DLGAP5 | up-regulated | 5.13E-15 | 1.37E-10 | 1.924044 |
| DPP3 | up-regulated | 4.43E-09 | 0.00011841 | 1.156478 |
| DSP | up-regulated | 1.31E-07 | 0.00349202 | 1.641113 |
| DTL | up-regulated | 2.11E-11 | 5.63E-07 | 1.866829 |
| E2F8 | up-regulated | 9.20E-10 | 2.46E-05 | 1.362166 |
| EDN2 | up-regulated | 1.93E-07 | 0.005150189 | 1.35261 |
| EFNA4 | up-regulated | 3.66E-09 | 9.79E-05 | 1.130629 |
| ELF3 | up-regulated | 4.67E-10 | 1.25E-05 | 1.452026 |
| ENC1 | up-regulated | 5.70E-08 | 0.001524363 | 1.118661 |
| EPN3 | up-regulated | 2.34E-07 | 0.006243171 | 1.408806 |
| EPYC | up-regulated | 8.01E-08 | 0.002142029 | 1.007072 |
| ERP27 | up-regulated | 1.20E-06 | 0.032164892 | 1.107111 |
| ESM1 | up-regulated | 2.37E-07 | 0.0063406 | 1.252224 |
| ESRP1 | up-regulated | 7.75E-08 | 0.002070706 | 1.576243 |
| EXO1 | up-regulated | 3.58E-11 | 9.57E-07 | 1.401094 |
| EZH2 | up-regulated | 2.36E-13 | 6.31E-09 | 1.906261 |
| EZR | up-regulated | 1.05E-09 | 2.81E-05 | 1.13575 |
| FADS2 | up-regulated | 3.59E-07 | 0.009597592 | 1.036286 |
| FAM110A | up-regulated | 2.75E-08 | 0.00073576 | 1.116068 |
| FAM83D | up-regulated | 4.02E-11 | 1.08E-06 | 1.980603 |
| FLVCR1 | up-regulated | 1.85E-10 | 4.95E-06 | 1.221648 |
| FN1 | up-regulated | 4.38E-14 | 1.17E-09 | 1.777832 |
| FOXM1 | up-regulated | 1.92E-12 | 5.13E-08 | 1.525332 |
| GALNT6 | up-regulated | 7.50E-08 | 0.002004335 | 1.573482 |
| GATA3 | up-regulated | 1.48E-07 | 0.003966694 | 1.146304 |
| GBP5 | up-regulated | 7.28E-10 | 1.94E-05 | 1.69312 |
| GINS1 | up-regulated | 1.38E-08 | 0.000369274 | 1.553029 |
| GINS2 | up-regulated | 1.39E-08 | 0.000371599 | 1.11223 |
| GJB2 | up-regulated | 2.03E-14 | 5.42E-10 | 2.012001 |
| GPRC5A | up-regulated | 1.03E-06 | 0.027404753 | 1.338681 |
| GRHL2 | up-regulated | 1.63E-08 | 0.000435481 | 1.264221 |
| HIST1H1B | up-regulated | 9.34E-12 | 2.50E-07 | 1.300161 |
| HIST1H2AI | up-regulated | 4.44E-07 | 0.011869181 | 1.208126 |
| HIST1H2BG | up-regulated | 8.81E-09 | 0.000235443 | 1.217092 |
| HIST1H3B | up-regulated | 6.48E-12 | 1.73E-07 | 1.473235 |
| HIST1H3D | up-regulated | 5.67E-09 | 0.000151666 | 1.432745 |
| HJURP | up-regulated | 4.16E-10 | 1.11E-05 | 1.5581 |
| HMGB3 | up-regulated | 1.31E-07 | 0.003495556 | 1.150328 |
| HMMR | up-regulated | 2.33E-09 | 6.22E-05 | 1.356226 |
| HN1 | up-regulated | 5.76E-11 | 1.54E-06 | 1.665661 |
| HOXC10 | up-regulated | 2.98E-08 | 0.000797452 | 1.103922 |
| HSD17B6 | up-regulated | 1.51E-09 | 4.05E-05 | 1.447698 |
| IBSP | up-regulated | 6.39E-07 | 0.017075019 | 1.004773 |
| IDH2 | up-regulated | 8.03E-07 | 0.021472506 | 1.036186 |
| IDO1 | up-regulated | 1.43E-06 | 0.038308561 | 1.132139 |
| IFI30 | up-regulated | 7.78E-11 | 2.08E-06 | 1.583688 |
| IFI6 | up-regulated | 9.01E-12 | 2.41E-07 | 1.505857 |
| IL4I1 | up-regulated | 1.23E-07 | 0.00329968 | 1.34185 |
| INHBA | up-regulated | 1.69E-15 | 4.51E-11 | 2.397096 |
| ISG15 | up-regulated | 3.14E-07 | 0.008380333 | 1.106871 |
| KCNK1 | up-regulated | 9.59E-11 | 2.56E-06 | 1.406818 |
| KIAA0101 | up-regulated | 5.74E-15 | 1.53E-10 | 2.107659 |
| KIF11 | up-regulated | 4.03E-14 | 1.08E-09 | 1.849112 |
| KIF14 | up-regulated | 4.67E-09 | 0.000124783 | 1.343295 |
| KIF15 | up-regulated | 8.66E-08 | 0.002314519 | 1.077634 |
| KIF18A | up-regulated | 2.32E-08 | 0.000618892 | 1.234303 |
| KIF20A | up-regulated | 5.20E-13 | 1.39E-08 | 1.666394 |
| KIF23 | up-regulated | 2.36E-08 | 0.00063104 | 1.332653 |
| KIF26B | up-regulated | 2.44E-08 | 0.000652811 | 1.038901 |
| KIF2C | up-regulated | 1.03E-11 | 2.74E-07 | 1.431716 |
| KIF4A | up-regulated | 5.74E-13 | 1.53E-08 | 1.601773 |
| KIFC1 | up-regulated | 1.79E-08 | 0.000478432 | 1.27578 |
| KNL1 | up-regulated | 9.75E-09 | 0.00026071 | 1.29523 |
| KPNA2 | up-regulated | 3.17E-08 | 0.000847938 | 1.101047 |
| KRT8 | up-regulated | 4.62E-07 | 0.012353754 | 1.444583 |
| LAMP3 | up-regulated | 1.32E-10 | 3.53E-06 | 1.52695 |
| LLGL2 | up-regulated | 8.08E-10 | 2.16E-05 | 1.165286 |
| LMNB1 | up-regulated | 1.80E-12 | 4.81E-08 | 1.645384 |
| LRP8 | up-regulated | 3.04E-08 | 0.000813056 | 1.115868 |
| LRRC59 | up-regulated | 3.01E-07 | 0.008042689 | 1.166502 |
| LSR | up-regulated | 4.68E-11 | 1.25E-06 | 1.488139 |
| MAL2 | up-regulated | 1.55E-07 | 0.004146856 | 1.043298 |
| MAPK13 | up-regulated | 3.01E-08 | 0.00080403 | 1.125228 |
| MB | up-regulated | 2.37E-08 | 0.000633852 | 1.335866 |
| MCM10 | up-regulated | 2.17E-08 | 0.000579315 | 1.115233 |
| MCM2 | up-regulated | 3.97E-08 | 0.001061395 | 1.054033 |
| MCM4 | up-regulated | 1.24E-08 | 0.000330251 | 1.23973 |
| MELK | up-regulated | 1.35E-12 | 3.61E-08 | 1.797973 |
| MFAP2 | up-regulated | 8.00E-09 | 0.000213829 | 1.027071 |
| MKI67 | up-regulated | 7.45E-13 | 1.99E-08 | 2.015322 |
| MMP1 | up-regulated | 2.83E-18 | 7.56E-14 | 3.079525 |
| MMP11 | up-regulated | 1.58E-20 | 4.22E-16 | 2.950357 |
| MMP12 | up-regulated | 5.78E-08 | 0.001544007 | 1.46464 |
| MMP13 | up-regulated | 2.66E-14 | 7.12E-10 | 2.152282 |
| MMP9 | up-regulated | 1.18E-12 | 3.15E-08 | 2.133666 |
| MND1 | up-regulated | 7.80E-07 | 0.020860786 | 1.016351 |
| MUC1 | up-regulated | 2.74E-08 | 0.000733642 | 1.527542 |
| MX1 | up-regulated | 8.89E-10 | 2.38E-05 | 1.136824 |
| MYBL2 | up-regulated | 1.37E-09 | 3.67E-05 | 1.557261 |
| NCAPG | up-regulated | 2.66E-10 | 7.11E-06 | 1.684577 |
| NDC80 | up-regulated | 1.04E-11 | 2.78E-07 | 1.439031 |
| NEK2 | up-regulated | 6.02E-11 | 1.61E-06 | 2.037443 |
| NFKBIE | up-regulated | 1.47E-06 | 0.03925214 | 1.021483 |
| NUF2 | up-regulated | 9.68E-16 | 2.59E-11 | 2.158974 |
| NUP210 | up-regulated | 1.02E-08 | 0.000272743 | 1.145684 |
| NUSAP1 | up-regulated | 4.32E-10 | 1.16E-05 | 1.775633 |
| OAS1 | up-regulated | 1.04E-07 | 0.002770276 | 1.103156 |
| OAS3 | up-regulated | 2.75E-07 | 0.007352133 | 1.054272 |
| OASL | up-regulated | 1.84E-07 | 0.004926878 | 1.067126 |
| OIP5 | up-regulated | 5.23E-08 | 0.001397487 | 1.240691 |
| ORC6 | up-regulated | 8.38E-08 | 0.002239969 | 1.087518 |
| P4HA3 | up-regulated | 3.80E-07 | 0.010158943 | 1.139633 |
| PAFAH1B3 | up-regulated | 1.39E-08 | 0.000372498 | 1.436755 |
| PBK | up-regulated | 4.44E-13 | 1.19E-08 | 1.959666 |
| PCDH17 | up-regulated | 1.19E-06 | 0.031700025 | 1.042628 |
| PKMYT1 | up-regulated | 1.21E-06 | 0.032221584 | 1.18472 |
| PPEF1 | up-regulated | 4.12E-10 | 1.10E-05 | 1.300463 |
| PRAME | up-regulated | 4.91E-09 | 0.000131218 | 1.279071 |
| PRC1 | up-regulated | 1.80E-09 | 4.80E-05 | 1.71285 |
| PTTG1 | up-regulated | 3.69E-12 | 9.87E-08 | 1.518988 |
| PYCR1 | up-regulated | 1.95E-08 | 0.00052217 | 1.274304 |
| RAB25 | up-regulated | 2.13E-10 | 5.68E-06 | 1.925462 |
| RACGAP1 | up-regulated | 1.30E-10 | 3.47E-06 | 1.343139 |
| RAD51 | up-regulated | 3.24E-10 | 8.67E-06 | 1.414802 |
| RAD51AP1 | up-regulated | 2.15E-08 | 0.000574348 | 1.265906 |
| RAD54L | up-regulated | 9.84E-07 | 0.026291505 | 1.076028 |
| RECQL4 | up-regulated | 9.98E-08 | 0.002667681 | 1.365288 |
| RGS1 | up-regulated | 1.39E-07 | 0.003710873 | 1.092351 |
| RRM2 | up-regulated | 1.21E-14 | 3.24E-10 | 2.484157 |
| RSAD2 | up-regulated | 1.02E-08 | 0.000272646 | 1.426897 |
| RTKN2 | up-regulated | 1.16E-08 | 0.000309491 | 1.059544 |
| S100A14 | up-regulated | 6.84E-09 | 0.000182716 | 1.749333 |
| S100A7 | up-regulated | 1.02E-07 | 0.002720402 | 1.42305 |
| S100P | up-regulated | 1.96E-12 | 5.25E-08 | 2.440204 |
| SDC1 | up-regulated | 1.30E-10 | 3.46E-06 | 1.950132 |
| SDS | up-regulated | 3.65E-09 | 9.77E-05 | 1.097477 |
| SHCBP1 | up-regulated | 3.83E-10 | 1.02E-05 | 1.181401 |
| SKA3 | up-regulated | 1.01E-07 | 0.002701537 | 1.108442 |
| SLAMF8 | up-regulated | 1.61E-08 | 0.000431593 | 1.369099 |
| SLC16A3 | up-regulated | 1.16E-06 | 0.031091638 | 1.232138 |
| SLC2A1 | up-regulated | 1.25E-07 | 0.003343688 | 1.036035 |
| SLC7A5 | up-regulated | 3.95E-07 | 0.01055283 | 1.131486 |
| SLC9A3R1 | up-regulated | 6.70E-07 | 0.017915743 | 1.066689 |
| SORD | up-regulated | 4.56E-07 | 0.012179751 | 1.111316 |
| SOX11 | up-regulated | 3.17E-08 | 0.000848382 | 1.606261 |
| SPAG5 | up-regulated | 1.18E-12 | 3.15E-08 | 1.692766 |
| SPC25 | up-regulated | 7.57E-12 | 2.02E-07 | 1.809803 |
| SPINT1 | up-regulated | 1.48E-08 | 0.000396114 | 1.112261 |
| SPP1 | up-regulated | 9.20E-11 | 2.46E-06 | 1.689762 |
| SQLE | up-regulated | 2.26E-09 | 6.04E-05 | 1.375565 |
| ST14 | up-regulated | 3.78E-09 | 0.000101166 | 1.106223 |
| SULF1 | up-regulated | 2.60E-12 | 6.95E-08 | 1.663057 |
| TACC3 | up-regulated | 9.14E-10 | 2.44E-05 | 1.25331 |
| TAP1 | up-regulated | 5.19E-10 | 1.39E-05 | 1.355937 |
| TDO2 | up-regulated | 6.69E-12 | 1.79E-07 | 1.680528 |
| TFAP2A | up-regulated | 1.59E-07 | 0.004252041 | 1.004731 |
| TIGIT | up-regulated | 2.16E-07 | 0.005762769 | 1.058078 |
| TK1 | up-regulated | 2.42E-11 | 6.48E-07 | 1.782685 |
| TLCD1 | up-regulated | 4.47E-08 | 0.001193596 | 1.055863 |
| TMEM132A | up-regulated | 1.48E-07 | 0.003966694 | 1.291737 |
| TNFRSF12A | up-regulated | 1.52E-07 | 0.004075317 | 1.109649 |
| TNFSF4 | up-regulated | 3.43E-08 | 0.000918055 | 1.239939 |
| TOP2A | up-regulated | 1.09E-14 | 2.92E-10 | 2.124486 |
| TPD52 | up-regulated | 1.75E-08 | 0.000467682 | 1.391983 |
| TPX2 | up-regulated | 1.07E-15 | 2.85E-11 | 2.045286 |
| TRIP13 | up-regulated | 9.01E-10 | 2.41E-05 | 1.337195 |
| TSTA3 | up-regulated | 1.76E-09 | 4.72E-05 | 1.405252 |
| TTK | up-regulated | 1.21E-14 | 3.23E-10 | 1.71213 |
| TUBB3 | up-regulated | 8.60E-08 | 0.002298896 | 1.283606 |
| TYMS | up-regulated | 1.00E-09 | 2.68E-05 | 1.320802 |
| UBD | up-regulated | 4.35E-13 | 1.16E-08 | 1.348427 |
| UBE2C | up-regulated | 4.26E-10 | 1.14E-05 | 1.47689 |
| UBE2S | up-regulated | 1.69E-07 | 0.004520404 | 1.080999 |
| UBE2T | up-regulated | 6.95E-14 | 1.86E-09 | 1.889621 |
| UHRF1 | up-regulated | 1.68E-11 | 4.48E-07 | 2.157144 |
| UNC5B | up-regulated | 2.58E-10 | 6.90E-06 | 1.204454 |
| WISP1 | up-regulated | 6.69E-09 | 0.000178789 | 1.326594 |
| ZWINT | up-regulated | 4.43E-07 | 0.011844849 | 1.161984 |
| AADAC | down-regulated | 6.07E-08 | 0.001623234 | -1.10996 |
| AASS | down-regulated | 6.51E-07 | 0.017388886 | -1.01185 |
| ABCA5 | down-regulated | 8.46E-07 | 0.022605083 | -1.13617 |
| ABCA6 | down-regulated | 1.02E-12 | 2.73E-08 | -2.42348 |
| ABCA8 | down-regulated | 3.43E-13 | 9.18E-09 | -2.56248 |
| ABCA9 | down-regulated | 1.70E-07 | 0.004546269 | -1.34796 |
| ABCD2 | down-regulated | 4.41E-07 | 0.011781834 | -1.23743 |
| ABI3BP | down-regulated | 4.73E-07 | 0.012631021 | -1.16763 |
| ACACB | down-regulated | 1.03E-07 | 0.002745792 | -1.58406 |
| ACADL | down-regulated | 1.76E-06 | 0.047022287 | -1.42733 |
| ACKR1 | down-regulated | 1.52E-07 | 0.0040517 | -1.29211 |
| ACO1 | down-regulated | 6.28E-09 | 0.000167754 | -1.20322 |
| ACSM5 | down-regulated | 2.03E-08 | 0.000542946 | -1.26618 |
| ACSS3 | down-regulated | 2.89E-09 | 7.72E-05 | -1.37024 |
| ACTG2 | down-regulated | 1.58E-08 | 0.000421536 | -1.52672 |
| ACVR1C | down-regulated | 4.53E-10 | 1.21E-05 | -2.14618 |
| ADAMTS1 | down-regulated | 6.23E-10 | 1.67E-05 | -1.36112 |
| ADAMTS5 | down-regulated | 1.33E-13 | 3.56E-09 | -2.17902 |
| ADD3 | down-regulated | 2.53E-11 | 6.76E-07 | -1.3564 |
| ADH1A | down-regulated | 1.24E-12 | 3.32E-08 | -2.16697 |
| ADH1B | down-regulated | 3.64E-19 | 9.72E-15 | -3.42672 |
| ADH1C | down-regulated | 9.62E-19 | 2.57E-14 | -3.19967 |
| ADH5 | down-regulated | 8.03E-10 | 2.15E-05 | -1.13249 |
| ADHFE1 | down-regulated | 2.58E-07 | 0.006902331 | -1.05276 |
| ADIPOQ | down-regulated | 7.91E-14 | 2.11E-09 | -3.18627 |
| ADM | down-regulated | 1.09E-06 | 0.029244818 | -1.11065 |
| ADRA2A | down-regulated | 7.70E-10 | 2.06E-05 | -1.50776 |
| ADRB2 | down-regulated | 2.02E-09 | 5.39E-05 | -1.25053 |
| AK5 | down-regulated | 1.17E-08 | 0.000311621 | -1.70995 |
| AKAP12 | down-regulated | 4.92E-09 | 0.000131439 | -1.67189 |
| AKR1C1 | down-regulated | 1.38E-14 | 3.69E-10 | -2.30749 |
| AKR1C3 | down-regulated | 1.68E-14 | 4.49E-10 | -2.4462 |
| ALDH1A1 | down-regulated | 4.26E-14 | 1.14E-09 | -2.39113 |
| ALDH1L1 | down-regulated | 4.46E-07 | 0.011914319 | -1.20698 |
| ALDH2 | down-regulated | 8.95E-09 | 0.000239357 | -1.41359 |
| AMOTL2 | down-regulated | 2.04E-09 | 5.46E-05 | -1.23533 |
| ANG | down-regulated | 1.43E-07 | 0.003821725 | -1.09185 |
| ANGPT1 | down-regulated | 8.77E-10 | 2.34E-05 | -1.53534 |
| ANGPTL1 | down-regulated | 9.91E-12 | 2.65E-07 | -2.61173 |
| ANGPTL2 | down-regulated | 4.58E-09 | 0.000122409 | -1.38798 |
| ANGPTL4 | down-regulated | 8.09E-11 | 2.16E-06 | -1.43811 |
| ANGPTL7 | down-regulated | 1.36E-07 | 0.003642471 | -1.81348 |
| ANKRD29 | down-regulated | 1.84E-10 | 4.91E-06 | -1.40261 |
| ANKRD35 | down-regulated | 1.25E-07 | 0.003331543 | -1.35958 |
| ANTXR2 | down-regulated | 6.40E-11 | 1.71E-06 | -1.40669 |
| ANXA1 | down-regulated | 3.71E-10 | 9.92E-06 | -1.72402 |
| ANXA3 | down-regulated | 2.73E-09 | 7.29E-05 | -1.24372 |
| AOC3 | down-regulated | 3.83E-10 | 1.02E-05 | -1.95952 |
| AOX1 | down-regulated | 1.34E-11 | 3.57E-07 | -1.76834 |
| APCDD1 | down-regulated | 9.22E-08 | 0.00246565 | -1.31692 |
| APOD | down-regulated | 2.08E-12 | 5.57E-08 | -2.01315 |
| AQP1 | down-regulated | 1.58E-08 | 0.000422002 | -1.05981 |
| AQP7 | down-regulated | 5.39E-09 | 0.000144164 | -1.57478 |
| AREG | down-regulated | 4.59E-07 | 0.012278161 | -1.57836 |
| ASPA | down-regulated | 5.53E-15 | 1.48E-10 | -2.15304 |
| ATOH8 | down-regulated | 2.60E-07 | 0.006944127 | -1.01211 |
| ATP1A2 | down-regulated | 1.16E-10 | 3.11E-06 | -3.02705 |
| BBOX1 | down-regulated | 1.73E-08 | 0.000461586 | -1.45653 |
| BCHE | down-regulated | 3.18E-07 | 0.008500452 | -1.05266 |
| BDH2 | down-regulated | 7.81E-08 | 0.002087727 | -1.01435 |
| BHMT2 | down-regulated | 8.37E-13 | 2.24E-08 | -1.89183 |
| BMP2 | down-regulated | 1.20E-06 | 0.032087011 | -1.00576 |
| BMPER | down-regulated | 1.31E-09 | 3.51E-05 | -1.35978 |
| C16orf89 | down-regulated | 2.40E-09 | 6.41E-05 | -1.53408 |
| C1orf115 | down-regulated | 1.06E-07 | 0.002832062 | -1.10601 |
| C1QTNF7 | down-regulated | 1.90E-07 | 0.005090026 | -1.16485 |
| C2orf40 | down-regulated | 9.22E-14 | 2.46E-09 | -2.14569 |
| C6 | down-regulated | 2.76E-09 | 7.37E-05 | -1.14954 |
| C7 | down-regulated | 2.58E-07 | 0.006902331 | -1.06391 |
| CA3 | down-regulated | 2.13E-09 | 5.69E-05 | -2.17625 |
| CA4 | down-regulated | 9.87E-09 | 0.000263726 | -1.70049 |
| CALB2 | down-regulated | 3.00E-07 | 0.008014449 | -1.52601 |
| CAPN6 | down-regulated | 1.50E-10 | 4.01E-06 | -2.17023 |
| CASQ2 | down-regulated | 5.96E-09 | 0.000159326 | -1.39931 |
| CAT | down-regulated | 1.29E-11 | 3.44E-07 | -1.43903 |
| CAV1 | down-regulated | 7.70E-15 | 2.06E-10 | -2.43533 |
| CAV2 | down-regulated | 1.61E-10 | 4.32E-06 | -1.83872 |
| CCDC3 | down-regulated | 7.06E-08 | 0.001887104 | -1.58394 |
| CCDC80 | down-regulated | 1.38E-08 | 0.000369274 | -1.52665 |
| CCL28 | down-regulated | 1.12E-08 | 0.000299675 | -1.51345 |
| CCNG1 | down-regulated | 5.55E-08 | 0.001483035 | -1.30018 |
| CD248 | down-regulated | 5.24E-10 | 1.40E-05 | -1.41003 |
| CD34 | down-regulated | 4.06E-07 | 0.010858519 | -1.1064 |
| CD36 | down-regulated | 5.40E-17 | 1.44E-12 | -2.92675 |
| CDKN1C | down-regulated | 5.54E-08 | 0.001479783 | -1.49961 |
| CDO1 | down-regulated | 7.70E-17 | 2.06E-12 | -3.07074 |
| CELF2 | down-regulated | 1.65E-07 | 0.00441816 | -1.06737 |
| CFD | down-regulated | 3.70E-14 | 9.88E-10 | -2.69504 |
| CFH | down-regulated | 1.79E-09 | 4.77E-05 | -1.38124 |
| CFL2 | down-regulated | 5.86E-11 | 1.57E-06 | -1.50017 |
| CHL1 | down-regulated | 2.03E-10 | 5.43E-06 | -1.395 |
| CHRDL1 | down-regulated | 3.51E-08 | 0.000939512 | -2.63009 |
| CIDEC | down-regulated | 4.73E-11 | 1.26E-06 | -2.70215 |
| CITED1 | down-regulated | 6.29E-08 | 0.001681584 | -2.05474 |
| CKMT2 | down-regulated | 1.66E-11 | 4.45E-07 | -1.86056 |
| CLDN5 | down-regulated | 1.22E-07 | 0.003255119 | -1.01932 |
| CLDN8 | down-regulated | 5.23E-09 | 0.000139877 | -1.34207 |
| CLEC3B | down-regulated | 6.17E-10 | 1.65E-05 | -1.73531 |
| CLIC2 | down-regulated | 2.93E-08 | 0.000782565 | -1.10211 |
| CLIP4 | down-regulated | 9.08E-10 | 2.43E-05 | -1.42534 |
| CLMP | down-regulated | 2.37E-07 | 0.0063406 | -1.48504 |
| CMA1 | down-regulated | 1.16E-09 | 3.10E-05 | -1.22081 |
| CNN1 | down-regulated | 2.18E-08 | 0.000581812 | -1.53818 |
| CNRIP1 | down-regulated | 1.24E-08 | 0.000332594 | -1.20474 |
| COL14A1 | down-regulated | 2.13E-09 | 5.69E-05 | -1.59783 |
| CPE | down-regulated | 6.93E-10 | 1.85E-05 | -1.64327 |
| CPED1 | down-regulated | 6.81E-07 | 0.018194585 | -1.3314 |
| CRIM1 | down-regulated | 4.86E-08 | 0.001299224 | -1.12666 |
| CRTAP | down-regulated | 1.48E-08 | 0.000394318 | -1.01589 |
| CRYAB | down-regulated | 9.05E-08 | 0.002418317 | -1.94749 |
| CRYBG3 | down-regulated | 2.57E-08 | 0.000685912 | -1.25054 |
| CSN1S1 | down-regulated | 3.83E-10 | 1.02E-05 | -1.50673 |
| CTNNAL1 | down-regulated | 2.03E-09 | 5.43E-05 | -1.37729 |
| CTSG | down-regulated | 8.36E-09 | 0.00022347 | -1.75179 |
| CTTNBP2 | down-regulated | 5.90E-09 | 0.000157685 | -1.39847 |
| CXCL12 | down-regulated | 7.81E-10 | 2.09E-05 | -1.84573 |
| CXCL14 | down-regulated | 7.75E-08 | 0.002072698 | -1.21001 |
| CXCL2 | down-regulated | 1.16E-10 | 3.10E-06 | -1.9155 |
| CYBRD1 | down-regulated | 3.05E-10 | 8.16E-06 | -1.433 |
| CYYR1 | down-regulated | 1.78E-10 | 4.74E-06 | -1.37295 |
| DCLK1 | down-regulated | 3.46E-09 | 9.25E-05 | -2.03032 |
| DCN | down-regulated | 3.56E-07 | 0.009518797 | -1.00909 |
| DDIT4L | down-regulated | 9.11E-08 | 0.002433697 | -1.08371 |
| DDR2 | down-regulated | 1.04E-06 | 0.027768832 | -1.41833 |
| DEFB132 | down-regulated | 1.35E-07 | 0.003609926 | -1.63085 |
| DENND2A | down-regulated | 1.95E-08 | 0.00052217 | -1.53142 |
| DGAT2 | down-regulated | 1.66E-06 | 0.044486723 | -1.72724 |
| DLC1 | down-regulated | 7.33E-10 | 1.96E-05 | -1.11663 |
| DLK1 | down-regulated | 1.82E-06 | 0.048554641 | -1.45282 |
| DMGDH | down-regulated | 5.72E-07 | 0.015288997 | -1.00634 |
| DNAJB4 | down-regulated | 1.61E-07 | 0.00430739 | -1.00857 |
| DNASE1L3 | down-regulated | 4.84E-08 | 0.001294321 | -1.25787 |
| DOCK11 | down-regulated | 8.33E-08 | 0.002226128 | -1.26606 |
| DPP4 | down-regulated | 9.22E-08 | 0.00246565 | -1.0806 |
| DPT | down-regulated | 5.46E-14 | 1.46E-09 | -2.38392 |
| DST | down-regulated | 1.36E-07 | 0.003642471 | -1.87625 |
| DUSP1 | down-regulated | 2.50E-09 | 6.68E-05 | -1.15643 |
| DUSP6 | down-regulated | 8.32E-09 | 0.000222371 | -1.0374 |
| EBF1 | down-regulated | 2.44E-10 | 6.53E-06 | -2.03677 |
| ECHDC1 | down-regulated | 2.96E-08 | 0.000790919 | -1.2562 |
| ECHDC3 | down-regulated | 7.24E-09 | 0.000193416 | -1.26125 |
| ECM2 | down-regulated | 4.40E-11 | 1.18E-06 | -1.59835 |
| EDNRB | down-regulated | 2.13E-11 | 5.70E-07 | -1.86857 |
| EFEMP1 | down-regulated | 8.34E-08 | 0.002228201 | -1.38015 |
| EGFLAM | down-regulated | 1.06E-07 | 0.00284179 | -1.21741 |
| EGR1 | down-regulated | 6.18E-12 | 1.65E-07 | -1.86481 |
| EGR2 | down-regulated | 2.89E-07 | 0.007729865 | -1.41835 |
| EGR3 | down-regulated | 1.42E-07 | 0.003799337 | -1.29697 |
| EHBP1 | down-regulated | 2.48E-08 | 0.000663936 | -1.02015 |
| EMCN | down-regulated | 1.45E-09 | 3.89E-05 | -1.47455 |
| EMP1 | down-regulated | 1.79E-09 | 4.77E-05 | -1.45252 |
| EMX2 | down-regulated | 1.00E-06 | 0.02671978 | -1.02062 |
| ENPP2 | down-regulated | 2.36E-13 | 6.31E-09 | -2.1396 |
| F10 | down-regulated | 4.76E-10 | 1.27E-05 | -1.54947 |
| F13A1 | down-regulated | 6.91E-09 | 0.000184824 | -1.57171 |
| F3 | down-regulated | 7.70E-10 | 2.06E-05 | -1.18119 |
| FABP4 | down-regulated | 1.23E-13 | 3.28E-09 | -3.11695 |
| FAM107A | down-regulated | 1.28E-10 | 3.43E-06 | -1.47981 |
| FAM13A | down-regulated | 1.05E-06 | 0.027986646 | -1.15766 |
| FAM162B | down-regulated | 1.68E-07 | 0.00449466 | -1.26485 |
| FAM3B | down-regulated | 1.13E-06 | 0.030165118 | -1.23026 |
| FAT4 | down-regulated | 1.36E-06 | 0.036444592 | -1.05631 |
| FAXDC2 | down-regulated | 2.69E-08 | 0.00071882 | -1.20553 |
| FBLN5 | down-regulated | 1.76E-10 | 4.70E-06 | -1.61374 |
| FERMT2 | down-regulated | 1.95E-07 | 0.005214868 | -1.27237 |
| FGF10 | down-regulated | 1.47E-07 | 0.003935316 | -1.14518 |
| FGF2 | down-regulated | 1.18E-13 | 3.15E-09 | -2.08674 |
| FGF7 | down-regulated | 1.25E-07 | 0.003331543 | -1.34375 |
| FGFBP2 | down-regulated | 2.52E-10 | 6.73E-06 | -1.64397 |
| FHL1 | down-regulated | 3.20E-15 | 8.55E-11 | -2.80497 |
| FIGF | down-regulated | 3.89E-14 | 1.04E-09 | -3.0282 |
| FLNC | down-regulated | 7.27E-08 | 0.001942661 | -1.15525 |
| FLRT2 | down-regulated | 1.73E-06 | 0.046250087 | -1.01477 |
| FMO2 | down-regulated | 1.23E-10 | 3.28E-06 | -1.92721 |
| FMOD | down-regulated | 4.60E-09 | 0.000123068 | -1.25837 |
| FOS | down-regulated | 1.06E-09 | 2.82E-05 | -1.64864 |
| FOSB | down-regulated | 1.00E-08 | 0.000267558 | -1.53622 |
| FOXN3 | down-regulated | 1.30E-08 | 0.000346788 | -1.00909 |
| FOXO1 | down-regulated | 3.81E-09 | 0.000101726 | -1.04983 |
| FREM1 | down-regulated | 3.87E-10 | 1.03E-05 | -2.36598 |
| FSTL1 | down-regulated | 1.31E-06 | 0.035121037 | -1.09155 |
| FXYD1 | down-regulated | 1.25E-10 | 3.34E-06 | -1.96849 |
| FZD4 | down-regulated | 2.23E-11 | 5.96E-07 | -1.61315 |
| G0S2 | down-regulated | 1.15E-16 | 3.08E-12 | -2.98655 |
| GABRP | down-regulated | 1.43E-07 | 0.003821725 | -1.27203 |
| GBE1 | down-regulated | 5.12E-09 | 0.000136904 | -1.1099 |
| GDF10 | down-regulated | 3.39E-10 | 9.05E-06 | -2.27644 |
| GGTA1P | down-regulated | 1.60E-07 | 0.004266019 | -1.6282 |
| GHR | down-regulated | 1.73E-09 | 4.63E-05 | -2.01277 |
| GLYAT | down-regulated | 9.22E-14 | 2.46E-09 | -2.47662 |
| GNAI1 | down-regulated | 7.86E-11 | 2.10E-06 | -1.68036 |
| GNG11 | down-regulated | 4.31E-11 | 1.15E-06 | -1.84405 |
| GPAM | down-regulated | 3.49E-09 | 9.34E-05 | -2.17241 |
| GPC3 | down-regulated | 1.35E-12 | 3.61E-08 | -2.52557 |
| GPD1 | down-regulated | 2.17E-10 | 5.81E-06 | -2.40904 |
| GPIHBP1 | down-regulated | 3.44E-08 | 0.000919497 | -1.36075 |
| GPR146 | down-regulated | 2.06E-07 | 0.005499988 | -1.5185 |
| GPR34 | down-regulated | 2.16E-07 | 0.005762559 | -1.39831 |
| GPRASP1 | down-regulated | 1.11E-06 | 0.029547781 | -1.45971 |
| GPX3 | down-regulated | 7.78E-11 | 2.08E-06 | -2.22607 |
| GSN | down-regulated | 1.16E-07 | 0.003104956 | -1.16995 |
| GSTM5 | down-regulated | 1.09E-09 | 2.92E-05 | -1.74962 |
| GULP1 | down-regulated | 7.46E-09 | 0.000199332 | -1.21648 |
| GYG2 | down-regulated | 5.64E-10 | 1.51E-05 | -2.07117 |
| HADH | down-regulated | 5.15E-09 | 0.00013763 | -1.13331 |
| HBA2 | down-regulated | 3.89E-07 | 0.010396727 | -2.0833 |
| HBB | down-regulated | 5.15E-08 | 0.001376054 | -2.03062 |
| HLF | down-regulated | 1.18E-13 | 3.15E-09 | -1.91751 |
| HOXA5 | down-regulated | 2.17E-09 | 5.80E-05 | -1.1526 |
| HOXA7 | down-regulated | 1.53E-11 | 4.09E-07 | -1.631 |
| HPSE2 | down-regulated | 3.20E-07 | 0.008555671 | -1.07391 |
| HRCT1 | down-regulated | 4.71E-07 | 0.012596074 | -1.11247 |
| HSD11B1 | down-regulated | 1.01E-08 | 0.00027014 | -1.2709 |
| HSD17B11 | down-regulated | 4.48E-09 | 0.000119663 | -1.36182 |
| HSPB2 | down-regulated | 4.13E-08 | 0.001104015 | -1.57216 |
| HSPB7 | down-regulated | 1.63E-12 | 4.35E-08 | -1.97109 |
| ID1 | down-regulated | 1.99E-07 | 0.005322149 | -1.06733 |
| ID4 | down-regulated | 5.97E-10 | 1.60E-05 | -1.18109 |
| IGF1 | down-regulated | 6.59E-14 | 1.76E-09 | -2.25742 |
| IGFBP6 | down-regulated | 3.11E-14 | 8.30E-10 | -2.20058 |
| IGSF10 | down-regulated | 6.27E-12 | 1.68E-07 | -2.30905 |
| IL33 | down-regulated | 2.38E-09 | 6.36E-05 | -1.79042 |
| IL6 | down-regulated | 1.12E-09 | 2.98E-05 | -1.28751 |
| ITGA7 | down-regulated | 3.26E-13 | 8.72E-09 | -2.02892 |
| ITIH5 | down-regulated | 2.47E-13 | 6.60E-09 | -2.33943 |
| ITM2A | down-regulated | 2.68E-11 | 7.16E-07 | -2.29759 |
| ITSN1 | down-regulated | 1.14E-08 | 0.000305694 | -1.07019 |
| JAM2 | down-regulated | 2.35E-09 | 6.29E-05 | -1.63733 |
| KANK1 | down-regulated | 1.12E-08 | 0.000299976 | -1.14833 |
| KBTBD11 | down-regulated | 1.55E-06 | 0.041479553 | -1.15499 |
| KCTD12 | down-regulated | 5.03E-08 | 0.001345648 | -1.00548 |
| KIT | down-regulated | 4.28E-10 | 1.14E-05 | -1.93115 |
| KLB | down-regulated | 3.39E-09 | 9.05E-05 | -1.81619 |
| KLF4 | down-regulated | 1.49E-10 | 3.99E-06 | -1.55229 |
| KLHL13 | down-regulated | 7.49E-09 | 0.000200333 | -1.74144 |
| KRT14 | down-regulated | 8.45E-08 | 0.002257532 | -1.0304 |
| LAMA2 | down-regulated | 1.62E-09 | 4.32E-05 | -1.67473 |
| LAMA4 | down-regulated | 1.87E-10 | 4.99E-06 | -1.28562 |
| LAMB3 | down-regulated | 6.90E-07 | 0.018442255 | -1.14888 |
| LAMC1 | down-regulated | 3.77E-10 | 1.01E-05 | -1.22118 |
| LARP6 | down-regulated | 5.31E-13 | 1.42E-08 | -1.68351 |
| LDB2 | down-regulated | 2.42E-10 | 6.46E-06 | -1.38622 |
| LDHB | down-regulated | 1.64E-09 | 4.38E-05 | -1.42031 |
| LEP | down-regulated | 5.40E-09 | 0.000144433 | -2.40572 |
| LGALS12 | down-regulated | 8.38E-08 | 0.002239969 | -1.83663 |
| LHFP | down-regulated | 1.60E-11 | 4.27E-07 | -1.97793 |
| LIFR | down-regulated | 1.61E-08 | 0.00042966 | -1.32544 |
| LINC00341 | down-regulated | 1.13E-07 | 0.003021938 | -1.11575 |
| LIPE | down-regulated | 1.53E-14 | 4.08E-10 | -2.43565 |
| LMOD1 | down-regulated | 1.02E-09 | 2.74E-05 | -1.13738 |
| LPAR1 | down-regulated | 5.36E-08 | 0.001432689 | -1.45285 |
| LPL | down-regulated | 2.45E-21 | 6.54E-17 | -3.90278 |
| LRFN5 | down-regulated | 1.02E-06 | 0.027156094 | -1.49432 |
| LRRN3 | down-regulated | 3.52E-09 | 9.41E-05 | -1.55691 |
| LRRN4CL | down-regulated | 2.05E-12 | 5.48E-08 | -2.26516 |
| LVRN | down-regulated | 5.94E-08 | 0.00158741 | -1.65892 |
| LYVE1 | down-regulated | 7.18E-17 | 1.92E-12 | -3.63277 |
| MAB21L1 | down-regulated | 3.56E-08 | 0.000952137 | -1.31288 |
| MAMDC2 | down-regulated | 2.29E-15 | 6.13E-11 | -2.64734 |
| MAOA | down-regulated | 3.43E-17 | 9.18E-13 | -2.76637 |
| MAOB | down-regulated | 2.96E-09 | 7.91E-05 | -1.24193 |
| MAPK10 | down-regulated | 1.63E-09 | 4.35E-05 | -1.13453 |
| MARC1 | down-regulated | 6.15E-08 | 0.001642984 | -1.57776 |
| MATN2 | down-regulated | 6.28E-11 | 1.68E-06 | -1.80807 |
| MEDAG | down-regulated | 1.51E-08 | 0.000402589 | -1.43332 |
| MEIS2 | down-regulated | 5.32E-10 | 1.42E-05 | -1.52755 |
| MEOX1 | down-regulated | 7.61E-09 | 0.000203361 | -1.23199 |
| MEOX2 | down-regulated | 2.87E-09 | 7.68E-05 | -1.25783 |
| MEST | down-regulated | 6.10E-10 | 1.63E-05 | -1.54511 |
| METTL7A | down-regulated | 2.78E-08 | 0.00074337 | -1.08594 |
| MFAP4 | down-regulated | 8.52E-13 | 2.28E-08 | -2.50729 |
| MGLL | down-regulated | 1.26E-08 | 0.000335705 | -1.41788 |
| MLXIPL | down-regulated | 1.22E-06 | 0.032478039 | -1.00207 |
| MMD | down-regulated | 3.81E-09 | 0.000101726 | -1.52948 |
| MME | down-regulated | 7.96E-16 | 2.13E-11 | -2.60729 |
| MMRN1 | down-regulated | 4.79E-10 | 1.28E-05 | -1.80482 |
| MRAP | down-regulated | 5.50E-09 | 0.000147127 | -1.72867 |
| MRGPRF | down-regulated | 4.96E-08 | 0.001326916 | -1.15343 |
| MT1M | down-regulated | 1.79E-09 | 4.77E-05 | -1.70001 |
| MTURN | down-regulated | 3.00E-08 | 0.000800743 | -1.49805 |
| MUCL1 | down-regulated | 5.23E-08 | 0.001397487 | -1.15269 |
| MYH11 | down-regulated | 1.08E-08 | 0.000287951 | -1.26691 |
| MYOC | down-regulated | 1.36E-09 | 3.65E-05 | -1.98533 |
| MYOM1 | down-regulated | 7.49E-10 | 2.00E-05 | -1.37227 |
| MYZAP | down-regulated | 1.19E-08 | 0.000317735 | -1.60152 |
| NAP1L5 | down-regulated | 1.50E-08 | 0.000401544 | -1.41884 |
| NAV3 | down-regulated | 1.78E-06 | 0.047640829 | -1.01326 |
| NDN | down-regulated | 5.86E-08 | 0.001565902 | -1.45169 |
| NDRG2 | down-regulated | 1.95E-07 | 0.005223624 | -1.21743 |
| NIPSNAP3B | down-regulated | 3.63E-07 | 0.009715067 | -1.14013 |
| NLGN1 | down-regulated | 3.13E-09 | 8.36E-05 | -1.84666 |
| NMT2 | down-regulated | 2.68E-11 | 7.15E-07 | -1.38152 |
| NNAT | down-regulated | 2.07E-09 | 5.52E-05 | -1.45077 |
| NOSTRIN | down-regulated | 8.01E-07 | 0.021417428 | -1.2166 |
| NPY1R | down-regulated | 1.60E-11 | 4.29E-07 | -2.54635 |
| NPY2R | down-regulated | 4.59E-07 | 0.012278161 | -1.70348 |
| NPY5R | down-regulated | 3.83E-08 | 0.001024271 | -1.19533 |
| NR2F1 | down-regulated | 2.86E-07 | 0.007651474 | -1.33167 |
| NR3C1 | down-regulated | 3.01E-10 | 8.03E-06 | -1.23971 |
| NR3C2 | down-regulated | 1.39E-09 | 3.72E-05 | -1.15982 |
| NRN1 | down-regulated | 1.03E-06 | 0.027563283 | -1.38498 |
| NTF4 | down-regulated | 1.24E-06 | 0.033207086 | -1.27478 |
| NTRK2 | down-regulated | 3.25E-10 | 8.69E-06 | -1.65398 |
| OGN | down-regulated | 3.79E-14 | 1.01E-09 | -3.61541 |
| OLFML1 | down-regulated | 1.15E-07 | 0.00306316 | -1.07315 |
| OMD | down-regulated | 7.13E-09 | 0.000190495 | -1.27925 |
| OSR1 | down-regulated | 2.04E-07 | 0.005458036 | -1.84996 |
| OXTR | down-regulated | 7.77E-09 | 0.000207746 | -2.22225 |
| P2RY12 | down-regulated | 8.95E-10 | 2.39E-05 | -1.09776 |
| P2RY14 | down-regulated | 2.77E-07 | 0.007396251 | -1.23709 |
| PALM | down-regulated | 3.60E-07 | 0.009631557 | -1.02979 |
| PALMD | down-regulated | 1.57E-13 | 4.20E-09 | -2.20451 |
| PAMR1 | down-regulated | 2.14E-09 | 5.72E-05 | -1.48196 |
| PCDH18 | down-regulated | 6.10E-12 | 1.63E-07 | -1.82138 |
| PCK1 | down-regulated | 2.19E-12 | 5.84E-08 | -2.66309 |
| PCOLCE2 | down-regulated | 3.06E-16 | 8.18E-12 | -3.41154 |
| PCSK5 | down-regulated | 5.52E-10 | 1.47E-05 | -1.22233 |
| PDE2A | down-regulated | 5.77E-12 | 1.54E-07 | -1.57721 |
| PDE8B | down-regulated | 1.30E-10 | 3.46E-06 | -1.66066 |
| PDGFD | down-regulated | 4.42E-10 | 1.18E-05 | -1.59289 |
| PDGFRL | down-regulated | 6.53E-09 | 0.00017442 | -1.35603 |
| PDK4 | down-regulated | 2.86E-12 | 7.63E-08 | -2.10811 |
| PDZD2 | down-regulated | 2.03E-08 | 0.000542946 | -1.20725 |
| PDZK1 | down-regulated | 8.78E-08 | 0.002347119 | -1.30961 |
| PDZRN3 | down-regulated | 1.63E-08 | 0.000434676 | -1.36926 |
| PI16 | down-regulated | 9.68E-11 | 2.59E-06 | -2.68827 |
| PID1 | down-regulated | 4.98E-10 | 1.33E-05 | -1.42596 |
| PIK3C2G | down-regulated | 2.32E-07 | 0.00621204 | -1.21401 |
| PLA2G16 | down-regulated | 2.28E-10 | 6.09E-06 | -1.66101 |
| PLA2G4A | down-regulated | 1.44E-11 | 3.85E-07 | -1.75015 |
| PLAGL1 | down-regulated | 2.26E-08 | 0.000604226 | -1.22496 |
| PLIN1 | down-regulated | 2.50E-13 | 6.69E-09 | -2.86083 |
| PLIN4 | down-regulated | 5.65E-11 | 1.51E-06 | -2.36752 |
| PLP1 | down-regulated | 4.73E-10 | 1.26E-05 | -1.78755 |
| PLPP1 | down-regulated | 2.32E-08 | 0.000618892 | -1.33114 |
| PLPP3 | down-regulated | 2.10E-07 | 0.005624615 | -1.38501 |
| PLSCR4 | down-regulated | 3.33E-12 | 8.89E-08 | -1.86828 |
| PPARG | down-regulated | 7.16E-15 | 1.91E-10 | -2.59958 |
| PPP1R14A | down-regulated | 1.11E-10 | 2.98E-06 | -1.79645 |
| PPP1R1A | down-regulated | 1.61E-13 | 4.30E-09 | -2.54437 |
| PRG4 | down-regulated | 1.55E-08 | 0.00041387 | -1.56526 |
| PRKAR2B | down-regulated | 3.70E-13 | 9.88E-09 | -2.0161 |
| PRNP | down-regulated | 1.53E-07 | 0.004076346 | -1.05017 |
| PROS1 | down-regulated | 1.19E-09 | 3.18E-05 | -1.64437 |
| PTGDS | down-regulated | 1.42E-08 | 0.000380201 | -1.08178 |
| PTGER3 | down-regulated | 4.43E-08 | 0.001184364 | -1.01908 |
| PTH1R | down-regulated | 1.79E-08 | 0.000478154 | -1.37429 |
| PTN | down-regulated | 1.23E-09 | 3.28E-05 | -2.12156 |
| PTX3 | down-regulated | 1.73E-06 | 0.046250087 | -1.06531 |
| RAI2 | down-regulated | 1.02E-07 | 0.00272693 | -1.28147 |
| RASD1 | down-regulated | 1.70E-10 | 4.55E-06 | -1.61429 |
| RASSF9 | down-regulated | 1.40E-09 | 3.74E-05 | -1.08953 |
| RBMS3 | down-regulated | 5.69E-09 | 0.000152054 | -1.24157 |
| RBP4 | down-regulated | 8.76E-14 | 2.34E-09 | -2.95007 |
| RBP7 | down-regulated | 1.09E-11 | 2.92E-07 | -1.96445 |
| RBPMS2 | down-regulated | 4.17E-07 | 0.01113818 | -1.12938 |
| RECK | down-regulated | 5.13E-08 | 0.00137065 | -1.51883 |
| RELN | down-regulated | 6.86E-08 | 0.001832914 | -1.33513 |
| RERGL | down-regulated | 3.33E-10 | 8.90E-06 | -1.99658 |
| RGL1 | down-regulated | 7.15E-07 | 0.019110827 | -1.03862 |
| RGN | down-regulated | 1.24E-08 | 0.000332594 | -1.12233 |
| RGS2 | down-regulated | 6.60E-10 | 1.76E-05 | -1.40256 |
| RHOBTB3 | down-regulated | 4.11E-09 | 0.000109847 | -1.08772 |
| RHOJ | down-regulated | 1.44E-07 | 0.003842903 | -1.26151 |
| RHOXF1 | down-regulated | 1.16E-06 | 0.03110871 | -1.13427 |
| RND3 | down-regulated | 8.88E-09 | 0.000237262 | -1.33352 |
| ROR1 | down-regulated | 1.56E-06 | 0.041590528 | -1.0763 |
| RSPO3 | down-regulated | 1.15E-13 | 3.08E-09 | -2.32019 |
| RUNX1T1 | down-regulated | 1.05E-10 | 2.81E-06 | -1.63416 |
| S100B | down-regulated | 2.30E-14 | 6.15E-10 | -2.67626 |
| S1PR1 | down-regulated | 2.12E-10 | 5.66E-06 | -1.23218 |
| SAA1 | down-regulated | 2.81E-07 | 0.007516071 | -1.66352 |
| SAA2 | down-regulated | 9.94E-08 | 0.002657709 | -1.83294 |
| SASH1 | down-regulated | 7.86E-08 | 0.002099831 | -1.05495 |
| SCARA5 | down-regulated | 7.61E-14 | 2.03E-09 | -2.94196 |
| SCN4B | down-regulated | 2.54E-09 | 6.80E-05 | -1.4345 |
| SDPR | down-regulated | 9.52E-15 | 2.54E-10 | -2.71186 |
| SEMA3C | down-regulated | 6.27E-07 | 0.016751269 | -1.32646 |
| SEMA3G | down-regulated | 1.74E-07 | 0.004652629 | -1.44245 |
| SEPP1 | down-regulated | 1.16E-08 | 0.000310043 | -1.57976 |
| SERINC1 | down-regulated | 1.50E-06 | 0.040154628 | -1.01279 |
| SESN1 | down-regulated | 1.40E-06 | 0.037422854 | -1.06854 |
| SFRP1 | down-regulated | 6.17E-13 | 1.65E-08 | -2.4662 |
| SGCE | down-regulated | 2.01E-09 | 5.36E-05 | -1.46571 |
| SGCG | down-regulated | 4.68E-11 | 1.25E-06 | -1.82382 |
| SGK2 | down-regulated | 7.42E-09 | 0.000198335 | -1.57002 |
| SH3BGRL2 | down-regulated | 4.22E-09 | 0.000112879 | -1.31587 |
| SH3D19 | down-regulated | 6.82E-08 | 0.001823524 | -1.58671 |
| SIK2 | down-regulated | 1.04E-08 | 0.000276687 | -1.22772 |
| SLC16A7 | down-regulated | 1.64E-08 | 0.000438369 | -1.62473 |
| SLC19A3 | down-regulated | 1.41E-15 | 3.77E-11 | -2.72787 |
| SLC22A3 | down-regulated | 5.55E-08 | 0.001483035 | -1.36021 |
| SLC7A10 | down-regulated | 4.22E-11 | 1.13E-06 | -1.74601 |
| SLIT2 | down-regulated | 3.58E-09 | 9.56E-05 | -1.38457 |
| SLIT3 | down-regulated | 1.45E-10 | 3.89E-06 | -1.28228 |
| SOBP | down-regulated | 1.70E-06 | 0.045541959 | -1.22252 |
| SOCS2 | down-regulated | 1.01E-07 | 0.002707875 | -1.25199 |
| SOD3 | down-regulated | 1.56E-10 | 4.17E-06 | -1.27758 |
| SORBS1 | down-regulated | 5.38E-12 | 1.44E-07 | -1.66722 |
| SOX17 | down-regulated | 1.39E-07 | 0.003718942 | -1.25514 |
| SPG20 | down-regulated | 1.71E-09 | 4.57E-05 | -1.0537 |
| SPRY1 | down-regulated | 5.56E-10 | 1.49E-05 | -1.24936 |
| SPRY2 | down-regulated | 3.77E-13 | 1.01E-08 | -2.21728 |
| SRPX | down-regulated | 2.01E-13 | 5.37E-09 | -2.61367 |
| SSPN | down-regulated | 1.58E-09 | 4.22E-05 | -1.34267 |
| SUCLA2 | down-regulated | 1.97E-08 | 0.000526656 | -1.1235 |
| SYNM | down-regulated | 3.98E-09 | 0.000106301 | -1.43999 |
| TCEAL7 | down-regulated | 1.56E-07 | 0.004170161 | -1.68707 |
| TCN1 | down-regulated | 9.42E-07 | 0.025172022 | -1.27664 |
| TESC | down-regulated | 8.23E-09 | 0.00022002 | -1.50904 |
| TF | down-regulated | 8.74E-09 | 0.000233576 | -1.40569 |
| TFPI | down-regulated | 7.35E-09 | 0.000196355 | -1.6067 |
| TFPI2 | down-regulated | 9.77E-10 | 2.61E-05 | -1.62476 |
| TGFBR2 | down-regulated | 5.48E-09 | 0.000146596 | -1.10061 |
| TGFBR3 | down-regulated | 1.07E-13 | 2.86E-09 | -2.15963 |
| THRA | down-regulated | 3.98E-08 | 0.001063146 | -1.11815 |
| THRSP | down-regulated | 3.85E-13 | 1.03E-08 | -2.47109 |
| TIMP4 | down-regulated | 2.01E-18 | 5.37E-14 | -4.24042 |
| TMEM100 | down-regulated | 1.15E-11 | 3.07E-07 | -2.26485 |
| TMEM220 | down-regulated | 3.39E-07 | 0.009049513 | -1.20184 |
| TMEM37 | down-regulated | 1.97E-07 | 0.005264242 | -1.30203 |
| TMEM55A | down-regulated | 3.25E-07 | 0.008676417 | -1.16311 |
| TMOD1 | down-regulated | 4.34E-12 | 1.16E-07 | -1.63644 |
| TMTC1 | down-regulated | 1.83E-12 | 4.89E-08 | -1.71008 |
| TNMD | down-regulated | 7.67E-12 | 2.05E-07 | -2.58408 |
| TNNT3 | down-regulated | 1.80E-09 | 4.80E-05 | -1.07788 |
| TNXB | down-regulated | 3.26E-11 | 8.72E-07 | -2.17008 |
| TPPP3 | down-regulated | 4.06E-07 | 0.010858519 | -1.03499 |
| TSHZ2 | down-regulated | 8.19E-10 | 2.19E-05 | -1.61542 |
| TSPAN7 | down-regulated | 7.28E-10 | 1.94E-05 | -1.58096 |
| TSPAN8 | down-regulated | 3.26E-08 | 0.000870432 | -1.22184 |
| TUSC5 | down-regulated | 6.32E-13 | 1.69E-08 | -2.3485 |
| TWIST2 | down-regulated | 3.10E-09 | 8.28E-05 | -1.43999 |
| TXNIP | down-regulated | 1.36E-08 | 0.000364839 | -1.22756 |
| UGP2 | down-regulated | 7.73E-07 | 0.020658957 | -1.10666 |
| VGLL3 | down-regulated | 5.68E-11 | 1.52E-06 | -1.83885 |
| VIT | down-regulated | 8.44E-12 | 2.25E-07 | -1.65857 |
| WIF1 | down-regulated | 1.35E-09 | 3.61E-05 | -2.2832 |
| WISP2 | down-regulated | 2.31E-09 | 6.19E-05 | -1.50284 |
| WNT11 | down-regulated | 5.27E-07 | 0.014098823 | -1.02792 |
| ZBTB16 | down-regulated | 4.26E-13 | 1.14E-08 | -1.91641 |
| ZCCHC24 | down-regulated | 4.32E-10 | 1.16E-05 | -1.60866 |
| ZFHX4 | down-regulated | 2.02E-08 | 0.000540593 | -1.05243 |
| ZFP36 | down-regulated | 6.51E-08 | 0.001741287 | -1.04391 |
| ZNF106 | down-regulated | 1.84E-07 | 0.004921511 | -1.0789 |
