# Supplementary material for: Identification of candidate biomarkers correlated with the pathogenesis and prognosis of breast cancer via integrated bioinformatics analysis
Source: Medicine (Baltimore). 2020 Dec 4;99(49):e23153. doi: 10.1097/MD.0000000000023153 (PMC7717725; doi:10.1097/MD.0000000000023153)
Supplement: Supplemental Digital Content [file medi-99-e23153-s002.docx]

Table S2. Information of GO enrichment analysis.

| Category | ID | Term | Genes | Count | adj*P*val |
| --- | --- | --- | --- | --- | --- |
| MF | GO:0008201 | heparin binding | FMOD, FGF7, FGF10, FSTL1, CXCL11, ABI3BP, CCL7, CXCL10, OGN, WISP2, WISP1, SAA1, ANG, RSPO3, COMP, CFH, PTN, FGF2, FN1, LPL, TNXB, CCDC80, ECM2, SOD3, SLIT2, SLIT3, PCOLCE2, SFRP1, CXCL13, CLEC3B, TGFBR3, ADAMTS1, EPYC, ADAMTS5, CTSG | 35 | 5.33E-14 |
| BP | GO:0051301 | cell division | KIFC1, GNAI1, NEK2, AURKA, NR3C1, PTTG1, CCNG1, LLGL2, CCNE2, FAM83D, SPC25, KIF2C, CDCA8, NCAPG, OIP5, CDCA2, SPG20, BUB1, SKA3, CCNA2, CDCA5, CDC7, KIF14, CDK1, CDC6, KIF11, NUF2, TPX2, CENPF, NDC80, CDC20, BIRC5, UBE2C, TACC3, CDC25C, CCNB1, CCNB2, SPAG5, KNL1, ZWINT, CKS2, BUB1B, UBE2S | 43 | 6.93E-08 |
| BP | GO:0007067 | mitotic nuclear division | NEK2, PKMYT1, ANLN, AURKA, NR3C1, CEP55, PTTG1, CCNG1, FAM83D, SPC25, KIF2C, OIP5, BUB1, CDCA2, SKA3, CCNA2, CDCA5, TUBB3, ASPM, CDK1, CDC6, KIF11, KIF15, NUF2, TPX2, CENPF, NDC80, BIRC5, CDC20, PBK, CDC25C, CCNB2, KNL1, KLHL13, BUB1B | 35 | 1.47E-07 |
| BP | GO:0030198 | extracellular matrix organization | IBSP, ELF3, DCN, VIT, DDR2, ABI3BP, LAMB3, HPSE2, COMP, FGF2, COL11A1, SPP1, FN1, COL10A1, RECK, CCDC80, SPINT1, ECM2, LAMA2, LAMA4, BGN, COL14A1, EGFLAM, FBLN5, ITGA7, MFAP2, LAMC1, JAM2 | 28 | 1.19E-05 |
| BP | GO:0008284 | positive regulation of cell proliferation | CLDN7, FGF7, PRC1, CD248, EDN2, FOXM1, PTH1R, TTK, ESM1, KIT, CXCL10, AKR1C3, EDNRB, HPSE2, ADRA2A, CEACAM6, PTN, RAB25, PDGFD, FGF2, DPP4, FN1, CDC7, KIF14, IL6, PRAME, TNFSF4, KLB, SOX11, TGFBR2, MLXIPL, LIFR, IGF1, CDC20, BIRC5, HOXC10, LEP, S100B, SFRP1, ADM, NTRK2, ID4, AREG, BAMBI, MAB21L1 | 45 | 4.68E-05 |
