# Supplementary material for: Identification of candidate biomarkers correlated with the pathogenesis and prognosis of breast cancer via integrated bioinformatics analysis
Source: Medicine (Baltimore). 2020 Dec 4;99(49):e23153. doi: 10.1097/MD.0000000000023153 (PMC7717725; doi:10.1097/MD.0000000000023153)
Supplement: Supplemental Digital Content [file medi-99-e23153-s003.docx]

Table S3. Information for pathway enrichment analysis.

| Term | Genes | Count | *P*-value |
| --- | --- | --- | --- |
| Epithelial-to-mesenchymal transition | AKAP12; ANGPTL2; BGN; CAV1; CHRDL1; COL14A1; CRYAB; CXCL12; CXCL13; CXCR4; DCN; DDR2; DPT; ECM2; EFEMP1; ENPP2; F13A1; FERMT2; FHL1; FLRT2; FN1; FSTL1; GNG11; IGF1; ITM2A; JAM2; LHFP; MEOX2; MFAP4; NR3C1; PDZRN3; PTGDS; PTX3; RECK; RGS2; RUNX1T1; SFRP1; SLIT2; SOBP; SRPX; SYNM; ZCCHC24; | 42 | 1.59E-22 |
| Cell Cycle, Mitotic | AURKA; BIRC5; BUB1; BUB1B; CCNA2; CCNB1; CCNB2; CCNE2; CDC20; CDC25C; CDC45; CDC6; CDC7; CDCA8; CDK1; CENPF; CENPM; CENPU; GINS1; GINS2; KIF18A; KIF20A; KIF23; KIF2C; CASC5; MCM10; MCM2; MCM4; MYBL2; NDC80; NEK2; NUF2; ORC6; PKMYT1; PRKAR2B; PTTG1; RRM2; SPC25; TOP2A; TYMS; UBE2C; ZWINT; | 42 | 1.23E-13 |
| Mesenchymal-to-epithelial transition | AP1M2; AREG; ATP2C2; C1orf106; CEACAM6; CLDN4; CLDN7; CYB561; DSP; ELF3; EPN3; ESRP1; EZR; GPRC5A; GRHL2; KCNK1; KRT8; LLGL2; LSR; MAPK13; MUC1; OAS1; RAB25; S100A14; S100P; SDC1; SLC9A3R1; SORD; SPINT1; ST14; TPD52; TSPAN8; | 32 | 1.27E-11 |
| G2/M DNA damage checkpoint | CCNB1; CDC25C; CDC45; CDC6; CDC7; CDK1; CHEK1; MCM10; MCM2; MCM4; ORC6; | 11 | 1.79E-09 |
| DNA Replication | BIRC5; BUB1; BUB1B; CDC20; CDC45; CDC6; CDC7; CDCA8; CDK1; CENPF; CENPM; CENPU; GINS1; GINS2; KIF18A; KIF20A; KIF23; KIF2C; CASC5; MCM10; MCM2; MCM4; NDC80; NEK2; NUF2; ORC6; PRKAR2B; SPC25; UBE2C; ZWINT; | 30 | 1.23E-08 |
| G2/M Checkpoints | CCNB1; CCNB2; CDC25C; CDC45; CDC6; CDC7; CDK1; CHEK1; MCM10; MCM2; MCM4; ORC6; | 12 | 1.79387E-08 |
| Mitotic M-M/G1 phases | BIRC5; BUB1; BUB1B; CDC20; CDC45; CDC6; CDC7; CDCA8; CDK1; CENPF; CENPM; CENPU; KIF18A; KIF20A; KIF23; KIF2C; CASC5; MCM10; MCM2; MCM4; NDC80; NEK2; NUF2; ORC6; PRKAR2B; SPC25; UBE2C; ZWINT; | 28 | 3.26175E-08 |
| Aurora B signaling | AURKA; BIRC5; BUB1; CDCA8; KIF20A; KIF23; KIF2C; KLHL13; NCAPG; NDC80; RACGAP1; | 11 | 1.11105E-07 |
| M Phase | BIRC5; BUB1; BUB1B; CDC20; CDCA8; CDK1; CENPF; CENPM; CENPU; KIF18A; KIF20A; KIF23; KIF2C; CASC5; NDC80; NEK2; NUF2; PRKAR2B; SPC25; UBE2C; ZWINT; | 21 | 1.65162E-07 |
| Integrin family cell surface interactions | ADM; ADRB2; BAMBI; BIRC5; BMP2; CAT; CAV1; CCNA2; CCNB1; CCNG1; CDK1; CHEK1; CHRDL1; CITED1; COL11A1; CPE; CTSG; CXCL12; CXCR4; DCN; DSP; DUSP1; DUSP6; EDN2; EDNRB; EGR1; EGR2; EGR3; F10; F13A1; FABP4; FIGF; FN1; FOS; FOSB; FOXO1; GATA3; GNAI1; GPAM; GSN; GULP1; IBSP; IGF1; IL6; ITGA7; ITSN1; JAM2; KLB; KLF4; KPNA2; KRT14; KRT8; LAMA2; LAMA4; LAMB3; LAMC1; LEP; MAPK10; MAPK13; MMP1; MMP12; MMP13; MMP9; NDRG2; NR3C1; NTRK2; PDGFD; PLA2G4A; PPARG; S100A7; S1PR1; SDC1; SESN1; SFRP1; SLC2A1; SLC9A3R1; SORBS1; SPP1; SPRY2; TAP1; TF; TGFBR2; TGFBR3; TK1; ZFP36; | 85 | 2.13365E-07 |
