# Supplementary material for: Identification of candidate biomarkers correlated with the pathogenesis and prognosis of breast cancer via integrated bioinformatics analysis
Source: Medicine (Baltimore). 2020 Dec 4;99(49):e23153. doi: 10.1097/MD.0000000000023153 (PMC7717725; doi:10.1097/MD.0000000000023153)
Supplement: Supplemental Digital Content [file medi-99-e23153-s004.docx]

Table S4. Information for PPI network.

| Name | Degree | MCODE Score | BetweennessCentrality | ClosenessCentrality |
| --- | --- | --- | --- | --- |
| CDK1 | 75 | 25.16129032 | 0.1524286 | 0.34534161 |
| CDC20 | 63 | 25.16129032 | 0.02048797 | 0.31376975 |
| CCNB1 | 60 | 25.16129032 | 0.03974394 | 0.32783019 |
| CCNA2 | 60 | 25.16129032 | 0.02137609 | 0.31306306 |
| CCNB2 | 59 | 25.16129032 | 0.01299822 | 0.31271091 |
| BUB1 | 58 | 25.16129032 | 0.01190397 | 0.31165919 |
| BUB1B | 53 | 25.16129032 | 0.00854652 | 0.30923248 |
| CDCA8 | 52 | 25.16129032 | 0.00820052 | 0.30650496 |
| TOP2A | 51 | 25.16129032 | 0.0112489 | 0.30923248 |
| KIF11 | 50 | 25.16129032 | 0.00596509 | 0.30583058 |
| BIRC5 | 48 | 25.38494624 | 0.00637432 | 0.30515917 |
| NDC80 | 47 | 25.16129032 | 0.0036525 | 0.30283224 |
| DLGAP5 | 47 | 25.16129032 | 0.00549598 | 0.30449069 |
| KIF2C | 47 | 25.16129032 | 0.00328103 | 0.30119177 |
| CENPF | 46 | 25.16129032 | 0.00368744 | 0.29989213 |
| ASPM | 46 | 25.16129032 | 0.00685839 | 0.30415755 |
| KIF20A | 45 | 25.16129032 | 0.004349 | 0.30349345 |
| UBE2C | 44 | 25.5816092 | 0.22353461 | 0.35145386 |
| AURKA | 43 | 25.16129032 | 0.01343769 | 0.31447964 |
| NCAPG | 43 | 25.74384236 | 0.00451068 | 0.29956897 |
| NUSAP1 | 43 | 25.16129032 | 0.00232116 | 0.30184582 |
| RRM2 | 42 | 24.42528736 | 0.01256738 | 0.30515917 |
| TPX2 | 41 | 24.19354839 | 0.01646293 | 0.31306306 |
| TTK | 41 | 25.38494624 | 0.00199447 | 0.30151844 |
| CEP55 | 40 | 24.19354839 | 0.00284004 | 0.2992465 |
| PBK | 39 | 25.38494624 | 0.00733158 | 0.29892473 |
| KIF4A | 36 | 25.86243386 | 0.00172883 | 0.2973262 |
| KIF23 | 35 | 24.92877493 | 0.001102 | 0.27148438 |
| MELK | 35 | 25.74384236 | 0.00129896 | 0.29796356 |
| NUF2 | 33 | 22.92333333 | 0.00215945 | 0.26964113 |
| KIF15 | 33 | 23.65811966 | 8.50E-04 | 0.27069133 |
| SPAG5 | 32 | 25.79365079 | 3.24E-04 | 0.29796356 |
| PRC1 | 31 | 21.84057971 | 0.0028772 | 0.29605964 |
| NEK2 | 30 | 24.85754986 | 0.0071064 | 0.30820399 |
| RACGAP1 | 29 | 19.91341991 | 0.01398511 | 0.27121951 |
| PTTG1 | 29 | 23.85230769 | 6.96E-04 | 0.29449153 |
| GNG11 | 29 | 21 | 0.01716459 | 0.26679463 |
| KIF18A | 28 | 18 | 5.65E-04 | 0.26911907 |
| HJURP | 28 | 19.91341991 | 9.53E-04 | 0.29293994 |
| CDC45 | 26 | 9.058823529 | 0.00858956 | 0.278 |
| CXCL12 | 26 | 21 | 0.0794777 | 0.29511677 |
| MKI67 | 26 | 23 | 9.26E-05 | 0.29263158 |
| LPAR1 | 26 | 21 | 0.03915612 | 0.27470356 |
| CHEK1 | 25 | 9.340659341 | 0.04953469 | 0.29171039 |
| GNAI1 | 25 | 21 | 0.01870529 | 0.26450999 |
| SAA1 | 25 | 21 | 0.01579979 | 0.26859903 |
| ANXA1 | 25 | 21 | 0.01789135 | 0.26937984 |
| CXCR4 | 24 | 21 | 0.01261789 | 0.26577438 |
| MCM4 | 23 | 10.58823529 | 0.00259414 | 0.27634195 |
| FOXM1 | 23 | 18.90952381 | 0.00134446 | 0.29263158 |
| CASC5 | 22 | 18 | 1.26E-04 | 0.26653883 |
| CDC6 | 22 | 9.991666667 | 0.00532715 | 0.29511677 |
| CXCL2 | 22 | 21 | 0.06035664 | 0.29956897 |
| S1PR1 | 22 | 21 | 0.00354229 | 0.26375712 |
| MCM2 | 21 | 9.230769231 | 0.00284024 | 0.27606753 |
| CXCL9 | 21 | 21 | 0 | 0.26350711 |
| CXCL10 | 21 | 21 | 0 | 0.26350711 |
| CXCL11 | 21 | 21 | 0 | 0.26350711 |
| CXCL13 | 21 | 21 | 0 | 0.26350711 |
| CCR7 | 21 | 21 | 0 | 0.26350711 |
| KIAA0101 | 21 | 20 | 0.02481285 | 0.29700855 |
| ADRA2A | 21 | 21 | 0 | 0.26350711 |
| NPY5R | 21 | 21 | 0 | 0.26350711 |
| NPY1R | 21 | 21 | 0 | 0.26350711 |
| CCL28 | 21 | 21 | 0 | 0.26350711 |
| PTGER3 | 21 | 21 | 0 | 0.26350711 |
| NPY2R | 21 | 21 | 0 | 0.26350711 |
| P2RY12 | 21 | 21 | 0 | 0.26350711 |
| P2RY14 | 21 | 21 | 0 | 0.26350711 |
| CENPU | 20 | 18 | 1.00E-04 | 0.26602871 |
| CENPM | 20 | 18 | 1.00E-04 | 0.26602871 |
| SPC25 | 19 | 18 | 1.04E-05 | 0.26577438 |
| ZWINT | 19 | 18 | 2.91E-04 | 0.26602871 |
| FN1 | 19 | 8 | 0.1079504 | 0.30752212 |
| CDCA5 | 18 | 18 | 0 | 0.26552053 |
| HMMR | 17 | 11.54285714 | 0.01078318 | 0.27524752 |
| TYMS | 16 | 10 | 0.00194541 | 0.29232387 |
| KIFC1 | 16 | 13 | 0.00106561 | 0.29049112 |
| CKS2 | 15 | 11.86813187 | 3.71E-04 | 0.2886812 |
| IL6 | 15 | 8 | 0.35429458 | 0.37415882 |
| MCM10 | 14 | 7.822222222 | 0.00310454 | 0.27174976 |
| EXO1 | 14 | 6.805555556 | 0.01026861 | 0.27201566 |
| IGF1 | 14 | 6 | 0.09005148 | 0.2880829 |
| OIP5 | 14 | 9 | 5.48E-05 | 0.25551471 |
| CDKN3 | 14 | 12 | 5.68E-06 | 0.26425856 |
| TACC3 | 13 | 11.86813187 | 1.00E-06 | 0.2640076 |
| PPARG | 13 | 2 | 0.11954969 | 0.30415755 |
| GPC3 | 13 | 8 | 0.04646761 | 0.30021598 |
| LAMC1 | 13 | 8 | 0.04689863 | 0.29764454 |
| ANLN | 12 | 10.71794872 | 2.23E-06 | 0.26275992 |
| PRKAR2B | 12 | 4.761904762 | 0.10124712 | 0.30549451 |
| EGR1 | 11 | 6 | 0.04455836 | 0.28958333 |
| DTL | 11 | 7.644444444 | 8.96E-05 | 0.27174976 |
| ADRB2 | 11 | 4 | 0.03234564 | 0.29293994 |
| DEPDC1 | 11 | 9.848484848 | 9.30E-07 | 0.2522686 |
| TF | 11 | 8 | 0.03117754 | 0.30250272 |
| RAD51 | 10 | 3 | 0.01341867 | 0.26964113 |
| ORC6 | 10 | 7.822222222 | 2.41E-05 | 0.27042802 |
| FOS | 10 | 3 | 0.11366734 | 0.31519274 |
| ALDH2 | 10 | 1.857142857 | 0.46567033 | 0.44827586 |
| FGF2 | 10 | 3 | 0.05039395 | 0.2650143 |
| MMP9 | 10 | 4 | 0.10299114 | 0.29232387 |
| CHRDL1 | 10 | 8 | 0.02958162 | 0.29700855 |
| GINS2 | 9 | 5.5 | 6.49E-04 | 0.26103286 |
| FABP4 | 9 | 2.2 | 0.01179757 | 0.25203989 |
| SDC1 | 9 | 3 | 0.02345046 | 0.2475512 |
| SPP1 | 9 | 8 | 0.00719424 | 0.29480382 |
| FSTL1 | 9 | 8 | 0.00692587 | 0.29511677 |
| KIF26B | 9 | 9 | 0 | 0.23883162 |
| CDC25C | 8 | 7 | 0.00204879 | 0.28367347 |
| CDC7 | 8 | 8 | 0 | 0.26859903 |
| ISG15 | 8 | 6 | 0.02163328 | 0.25716929 |
| OAS1 | 8 | 6 | 0.00474422 | 0.23322148 |
| OAS3 | 8 | 6 | 0.00474422 | 0.23322148 |
| LPL | 8 | 3 | 0.04980324 | 0.24711111 |
| KPNA2 | 8 | 6 | 2.81E-05 | 0.26054358 |
| E2F8 | 8 | 8 | 0 | 0.24536628 |
| AOX1 | 8 | 3 | 0.43643956 | 0.44067797 |
| TMEM132A | 8 | 8 | 0 | 0.29449153 |
| MX1 | 7 | 6 | 7.42E-06 | 0.23302598 |
| OASL | 7 | 5 | 0.00474422 | 0.23302598 |
| BLM | 7 | 3.733333333 | 0.0014342 | 0.23781009 |
| RSAD2 | 7 | 6 | 7.42E-06 | 0.23302598 |
| IFI6 | 7 | 6 | 7.42E-06 | 0.23302598 |
| ADIPOQ | 7 | 2.7 | 0.02877119 | 0.29892473 |
| CFD | 7 | 6 | 0.01433655 | 0.24977538 |
| EDNRB | 7 | 6 | 3.69E-05 | 0.22401289 |
| NR3C1 | 7 | 3 | 0.02715984 | 0.25812442 |
| MMP1 | 7 | 1.2 | 0.02133566 | 0.25135624 |
| DCN | 7 | 3 | 0.01517431 | 0.25457875 |
| F13A1 | 7 | 6 | 0.0214269 | 0.25 |
| LAMB3 | 7 | 4 | 0.0169364 | 0.23720137 |
| NUP210 | 7 | 6 | 1.75E-05 | 0.26152399 |
| KLHL13 | 7 | 3.238095238 | 0.00831411 | 0.27121951 |
| CCNE2 | 6 | 3.733333333 | 2.80E-05 | 0.26005613 |
| EDN2 | 6 | 6 | 0 | 0.22329317 |
| SHCBP1 | 6 | 4.761904762 | 5.19E-06 | 0.24471831 |
| MAOA | 6 | 2.2 | 0.17491575 | 0.44827586 |
| FIGF | 6 | 6 | 0 | 0.24932735 |
| COL11A1 | 6 | 2.7 | 0.01215011 | 0.20115774 |
| PROS1 | 6 | 6 | 0 | 0.24932735 |
| COL14A1 | 6 | 2.7 | 0.0269258 | 0.23032312 |
| MMRN1 | 6 | 6 | 0 | 0.24932735 |
| OXTR | 6 | 6 | 0 | 0.22329317 |
| UBE2S | 5 | 4 | 1.29E-04 | 0.26990291 |
| GINS1 | 5 | 5 | 0 | 0.2192429 |
| HBB | 5 | 4 | 0.00719424 | 0.22730989 |
| PLIN1 | 5 | 1.666666667 | 0.02794384 | 0.23720137 |
| ADH1B | 5 | 2.4 | 0.03071062 | 0.41935484 |
| GPIHBP1 | 5 | 1.066666667 | 0.01434953 | 0.1991404 |
| LMNB1 | 5 | 2.7 | 0.0071961 | 0.26029963 |
| MYBL2 | 5 | 4 | 0.00896419 | 0.26054358 |
| TRIP13 | 5 | 4 | 1.47E-04 | 0.26275992 |
| LEP | 5 | 3 | 0.03635196 | 0.30449069 |
| KIF14 | 5 | 5 | 0 | 0.24027658 |
| SKA3 | 5 | 5 | 0 | 0.25090253 |
| FZD4 | 5 | 3 | 0.00490531 | 0.26325758 |
| BRIP1 | 5 | 3.733333333 | 3.10E-04 | 0.22768223 |
| LAMA2 | 5 | 4 | 1.01E-04 | 0.23283082 |
| DUSP1 | 5 | 3 | 0.01436252 | 0.24385965 |
| LAMA4 | 5 | 4 | 1.01E-04 | 0.23283082 |
| MAOB | 5 | 3 | 0.15512088 | 0.44067797 |
| ZBTB16 | 5 | 4 | 0.00602993 | 0.26937984 |
| MMP13 | 5 | 1.666666667 | 0.01784538 | 0.23559322 |
| IL4I1 | 5 | 2 | 0.22153846 | 0.34210526 |
| EZR | 4 | 4 | 0 | 0.26602871 |
| SLC9A3R1 | 4 | 4 | 0 | 0.26602871 |
| RAD51AP1 | 4 | 4 | 0 | 0.21517028 |
| ANGPTL4 | 4 | 3 | 0.00819813 | 0.23679727 |
| ACTG2 | 4 | 3 | 0.5 | 1 |
| ADH1A | 4 | 2.7 | 0.02373626 | 0.3880597 |
| CAV1 | 4 | 0.4 | 0.01664916 | 0.19814683 |
| UBE2T | 4 | 1.2 | 0.00264433 | 0.26911907 |
| ADH1C | 4 | 2.7 | 0.02373626 | 0.3880597 |
| BMP2 | 4 | 1.666666667 | 0.00727216 | 0.2312812 |
| ITSN1 | 4 | 3 | 0.01095296 | 0.2612782 |
| RHOJ | 4 | 0.4 | 0.01684729 | 0.25249773 |
| CTSG | 4 | 1.666666667 | 0.0087456 | 0.24711111 |
| EZH2 | 4 | 2 | 0.07839672 | 0.29293994 |
| BGN | 4 | 3 | 0.00176438 | 0.23986195 |
| BAMBI | 4 | 1.2 | 0.01644647 | 0.23244147 |
| PTX3 | 4 | 4 | 0 | 0.22712418 |
| GBP5 | 4 | 3 | 7.42E-06 | 0.18976109 |
| IFI30 | 4 | 3 | 7.42E-06 | 0.18976109 |
| COL10A1 | 4 | 2.7 | 1.73E-05 | 0.19028063 |
| KIT | 4 | 0.4 | 0.04958642 | 0.27389163 |
| FGF10 | 4 | 3 | 2.60E-05 | 0.2101285 |
| EBF1 | 4 | 2.7 | 4.18E-04 | 0.25551471 |
| UHRF1 | 4 | 4 | 0 | 0.24450308 |
| TFAP2A | 4 | 0.4 | 0.01278736 | 0.24152911 |
| P4HA3 | 4 | 3 | 0.00719424 | 0.19028063 |
| TCN1 | 4 | 4 | 0 | 0.22712418 |
| METTL7A | 4 | 4 | 0 | 0.22712418 |
| PRNP | 4 | 4 | 0 | 0.23263598 |
| TK1 | 3 | 1.666666667 | 2.16E-06 | 0.25812442 |
| LRP8 | 3 | 2 | 0.01433655 | 0.23781009 |
| AKR1C3 | 3 | 0.5 | 0.15076923 | 0.26804124 |
| F10 | 3 | 2 | 0.01433655 | 0.20072202 |
| CAT | 3 | 0.5 | 0.21846154 | 0.33766234 |
| CDKN1C | 3 | 3 | 0 | 0.25932836 |
| MYH11 | 3 | 3 | 0 | 0.8 |
| RAD54L | 3 | 3 | 0 | 0.21500387 |
| TGFBR2 | 3 | 2 | 0.00526057 | 0.19674452 |
| GATA3 | 3 | 0.5 | 0.01285321 | 0.2432196 |
| SOCS2 | 3 | 2 | 0.00719424 | 0.21401078 |
| ITGA7 | 3 | 3 | 0 | 0.2299421 |
| LMOD1 | 3 | 3 | 0 | 0.8 |
| HIST1H2BG | 3 | 2 | 0.66666667 | 1 |
| MAPK10 | 3 | 3 | 0 | 0.24343257 |
| MFAP2 | 3 | 3 | 0 | 1 |
| FBLN5 | 3 | 3 | 0 | 1 |
| MFAP4 | 3 | 3 | 0 | 1 |
| HSD17B6 | 3 | 0.5 | 0.28 | 0.34210526 |
| AOC3 | 3 | 3 | 0 | 0.37142857 |
| SLIT2 | 3 | 2 | 0.00719424 | 0.22880658 |
| ADH5 | 3 | 0.5 | 0.10065934 | 0.3880597 |
| CLDN4 | 3 | 3 | 0 | 1 |
| CLDN7 | 3 | 3 | 0 | 1 |
| EFEMP1 | 3 | 3 | 0 | 1 |
| FGF7 | 3 | 3 | 0 | 0.20996979 |
| FGFBP2 | 3 | 3 | 0 | 0.20996979 |
| TPD52 | 3 | 2 | 0.66666667 | 1 |
| PTH1R | 3 | 3 | 0 | 0.24006908 |
| CRTAP | 3 | 3 | 0 | 0.19002051 |
| ADM | 3 | 3 | 0 | 0.24006908 |
| SORBS1 | 3 | 3 | 0 | 0.8 |
| CLDN8 | 3 | 3 | 0 | 1 |
| CLDN5 | 3 | 3 | 0 | 1 |
| NTRK2 | 2 | 0.666666667 | 0.00719424 | 0.22419355 |
| RELN | 2 | 0.666666667 | 0.00719424 | 0.19252078 |
| LIPE | 2 | 2 | 0 | 0.20262391 |
| CFB | 2 | 0.666666667 | 0.00719424 | 0.20028818 |
| F3 | 2 | 2 | 0 | 0.16736905 |
| TFPI | 2 | 2 | 0 | 0.16736905 |
| SOD3 | 2 | 0.666666667 | 0.07692308 | 0.26 |
| FOSB | 2 | 2 | 0 | 0.2522686 |
| TGFBR3 | 2 | 2 | 0 | 0.19198895 |
| HIST1H3B | 2 | 2 | 0 | 0.75 |
| HIST1H3D | 2 | 2 | 0 | 0.75 |
| WNT11 | 2 | 2 | 0 | 0.22081017 |
| HOXA7 | 2 | 0.666666667 | 0.00719424 | 0.24027658 |
| S100B | 2 | 0.666666667 | 0.00980942 | 0.23619371 |
| FAM83D | 2 | 2 | 0 | 0.23924269 |
| CD36 | 2 | 2 | 0 | 0.23380992 |
| OMD | 2 | 2 | 0 | 1 |
| OGN | 2 | 2 | 0 | 1 |
| GYG2 | 2 | 0.666666667 | 1 | 1 |
| EGR2 | 2 | 0.666666667 | 0.00719424 | 0.22712418 |
| PCK1 | 2 | 2 | 0 | 0.23380992 |
| MUC1 | 2 | 2 | 0 | 1 |
| GALNT6 | 2 | 2 | 0 | 1 |
| MLXIPL | 2 | 0.666666667 | 0.00215462 | 0.27524752 |
| HPSE2 | 2 | 2 | 0 | 0.23699915 |
| CHST11 | 2 | 2 | 0 | 0.20321637 |
| FMOD | 2 | 2 | 0 | 1 |
| MUCL1 | 2 | 2 | 0 | 1 |
| DGAT2 | 2 | 0.666666667 | 0.00719424 | 0.16616856 |
| ACSS3 | 2 | 0.666666667 | 0.0410989 | 0.32911392 |
| RECQL4 | 2 | 2 | 0 | 0.21786834 |
| SSPN | 2 | 0.666666667 | 1 | 1 |
| HADH | 2 | 0.666666667 | 0.03802198 | 0.325 |
| ECHDC1 | 2 | 0.666666667 | 0.00835165 | 0.28888889 |
| ESRP1 | 2 | 0.666666667 | 0.00719424 | 0.23579304 |
| CITED1 | 2 | 0.666666667 | 0.00319222 | 0.22564935 |
| FADS2 | 2 | 0.666666667 | 1 | 1 |
| PPAP2B | 2 | 0.666666667 | 0.01433655 | 0.19885551 |
| ADAMTS5 | 2 | 0.666666667 | 0.00719424 | 0.19106529 |
| PCSK5 | 2 | 2 | 0 | 0.19871337 |
| CDCA2 | 2 | 2 | 0 | 0.23185988 |
| NRN1 | 2 | 2 | 0 | 0.16626794 |
| PIK3C2G | 2 | 2 | 0 | 0.2094951 |
| FLRT2 | 2 | 2 | 0 | 0.20981132 |
| AP1M2 | 2 | 2 | 0 | 0.75 |
| SH3D19 | 2 | 2 | 0 | 0.75 |
| RECK | 2 | 2 | 0 | 0.16626794 |
| MND1 | 1 | 0 | 0 | 0.21253823 |
| NTF4 | 1 | 0 | 0 | 0.18325643 |
| SPINT1 | 1 | 0 | 0 | 1 |
| ST14 | 1 | 0 | 0 | 1 |
| AKR1C1 | 1 | 0 | 0 | 0.21311475 |
| HBA2 | 1 | 0 | 0 | 0.18533333 |
| IGFBP6 | 1 | 0 | 0 | 0.22383253 |
| RGS1 | 1 | 0 | 0 | 0.20933735 |
| CAV2 | 1 | 0 | 0 | 0.16547619 |
| IL33 | 1 | 0 | 0 | 0.19577465 |
| HOXA5 | 1 | 0 | 0 | 0.19386332 |
| ACVR1C | 1 | 0 | 0 | 1 |
| INHBA | 1 | 0 | 0 | 1 |
| GHR | 1 | 0 | 0 | 0.17639594 |
| IDH2 | 1 | 0 | 0 | 1 |
| ACO1 | 1 | 0 | 0 | 1 |
| GPX3 | 1 | 0 | 0 | 0.208 |
| BMPER | 1 | 0 | 0 | 0.18796484 |
| HIST1H2AI | 1 | 0 | 0 | 0.6 |
| GBE1 | 1 | 0 | 0 | 0.66666667 |
| EGR3 | 1 | 0 | 0 | 0.18520986 |
| MGLL | 1 | 0 | 0 | 0.19828816 |
| FOXO1 | 1 | 0 | 0 | 0.27254902 |
| LYVE1 | 1 | 0 | 0 | 0.21600622 |
| CMA1 | 1 | 0 | 0 | 0.20101229 |
| ANGPT1 | 1 | 0 | 0 | 0.23539373 |
| GPD1 | 1 | 0 | 0 | 1 |
| GPAM | 1 | 0 | 0 | 1 |
| SORD | 1 | 0 | 0 | 0.25490196 |
| CFH | 1 | 0 | 0 | 0.16696697 |
| HSD11B1 | 1 | 0 | 0 | 0.2334173 |
| ZFP36 | 1 | 0 | 0 | 0.19618913 |
| SPRY1 | 1 | 0 | 0 | 1 |
| SPRY2 | 1 | 0 | 0 | 1 |
| NR3C2 | 1 | 0 | 0 | 0.20531758 |
| PTGDS | 1 | 0 | 0 | 0.21311475 |
| KRT8 | 1 | 0 | 0 | 1 |
| KRT14 | 1 | 0 | 0 | 1 |
| NOSTRIN | 1 | 0 | 0 | 0.16547619 |
| UGP2 | 1 | 0 | 0 | 0.66666667 |
| FXYD1 | 1 | 0 | 0 | 1 |
| ATP1A2 | 1 | 0 | 0 | 1 |
| PAFAH1B3 | 1 | 0 | 0 | 0.16153399 |
| TNNT3 | 1 | 0 | 0 | 1 |
| TMOD1 | 1 | 0 | 0 | 1 |
| CIDEC | 1 | 0 | 0 | 0.19185645 |
| SEPP1 | 1 | 0 | 0 | 1 |
| CLEC3B | 1 | 0 | 0 | 1 |
| MAL2 | 1 | 0 | 0 | 0.6 |
| CD34 | 1 | 0 | 0 | 0.21517028 |
| MME | 1 | 0 | 0 | 1 |
| DPP4 | 1 | 0 | 0 | 1 |
| PPAP2A | 1 | 0 | 0 | 0.1425641 |
| PCOLCE2 | 1 | 0 | 0 | 0.16757083 |
| IBSP | 1 | 0 | 0 | 0.22786885 |
| BBOX1 | 1 | 0 | 0 | 0.31325301 |
| C7 | 1 | 0 | 0 | 1 |
| C6 | 1 | 0 | 0 | 1 |
| CEACAM6 | 1 | 0 | 0 | 0.23539373 |
| DLC1 | 1 | 0 | 0 | 0.20174165 |
| CXCL14 | 1 | 0 | 0 | 0.2101285 |
| SGCE | 1 | 0 | 0 | 0.66666667 |
| DST | 1 | 0 | 0 | 0.19185645 |
| ADHFE1 | 1 | 0 | 0 | 0.2826087 |
| ALDH1A1 | 1 | 0 | 0 | 0.25742574 |
| KLB | 1 | 0 | 0 | 0.20965309 |
| ACSM5 | 1 | 0 | 0 | 0.66666667 |
| DUSP6 | 1 | 0 | 0 | 0.19618913 |
| ADAMTS1 | 1 | 0 | 0 | 0.16050808 |
| RASD1 | 1 | 0 | 0 | 0.20933735 |
| MMP12 | 1 | 0 | 0 | 0.19185645 |
| S100A7 | 1 | 0 | 0 | 0.19828816 |
| SGCG | 1 | 0 | 0 | 0.66666667 |
| FAM13A | 1 | 0 | 0 | 0.20174165 |
| ENPP2 | 1 | 0 | 0 | 0.21567106 |
| HIST1H1B | 1 | 0 | 0 | 0.20669145 |
| MMP11 | 1 | 0 | 0 | 0.20101229 |
| RBPMS2 | 1 | 0 | 0 | 0.19093407 |
| DPT | 1 | 0 | 0 | 0.20306793 |
| SLIT3 | 1 | 0 | 0 | 0.18632708 |
| CNN1 | 1 | 0 | 0 | 0.57142857 |
| TDO2 | 1 | 0 | 0 | 0.25742574 |
| MRAP | 1 | 0 | 0 | 0.20965309 |
| AK5 | 1 | 0 | 0 | 0.23400673 |
| ATAD2 | 1 | 0 | 0 | 0.19467787 |
| PYCR1 | 1 | 0 | 0 | 0.15995397 |
| BHMT2 | 1 | 0 | 0 | 0.25742574 |
| ASPA | 1 | 0 | 0 | 0.25742574 |
| PLA2G16 | 1 | 0 | 0 | 0.66666667 |
