# Supplementary material for: Identification of candidate biomarkers correlated with the pathogenesis and prognosis of breast cancer via integrated bioinformatics analysis
Source: Medicine (Baltimore). 2020 Dec 4;99(49):e23153. doi: 10.1097/MD.0000000000023153 (PMC7717725; doi:10.1097/MD.0000000000023153)
Supplement: Supplemental Digital Content [file medi-99-e23153-s005.docx]

Table S5. Information for module 1 and module 2.

| MCODE Cluster | Pvalue | Count | Description |
| --- | --- | --- | --- |
| Module 1 | 8.28814E-29 | 21 | Cell Cycle, Mitotic |
| Module 1 | 1.15413E-20 | 14 | M Phase |
| Module 1 | 3.65671E-19 | 12 | PLK1 signaling events |
| Module 1 | 6.59064E-19 | 12 | Polo-like kinase signaling events in the cell cycle |
| Module 1 | 5.08501E-18 | 14 | Mitotic M-M/G1 phases |
| Module 1 | 1.4766E-17 | 14 | DNA Replication |
| Module 1 | 1.8264E-17 | 11 | Signaling by Aurora kinases |
| Module 1 | 3.20225E-17 | 9 | Aurora B signaling |
| Module 1 | 1.86658E-15 | 10 | Mitotic Prometaphase |
| Module 1 | 1.11569E-12 | 7 | FOXM1 transcription factor network |
| Module 2 | 6.16692E-28 | 17 | Class A/1 (Rhodopsin-like receptors) |
| Module 2 | 2.14118E-26 | 17 | GPCR ligand binding |
| Module 2 | 7.23911E-22 | 18 | Signaling by GPCR |
| Module 2 | 1.18289E-18 | 18 | Signal Transduction |
| Module 2 | 9.86286E-18 | 11 | Peptide ligand-binding receptors |
| Module 2 | 2.33084E-14 | 7 | Chemokine receptors bind chemokines |
| Module 2 | 5.11874E-10 | 5 | CXCR3-mediated signaling events |
| Module 2 | 4.658E-06 | 3 | S1P3 pathway |
| Module 2 | 1.86893E-05 | 2 | ADP signalling through P2Y purinoceptor 12 |
| Module 2 | 3.48383E-05 | 2 | Lysosphingolipid and LPA receptors |
